# A Genetic Screening Strategy Identifies Novel Regulators of the Proteostasis Network

**DOI:** 10.1371/journal.pgen.1002438

**Published:** 2011-12-29

**Authors:** M. Catarina Silva, Susan Fox, Monica Beam, Happy Thakkar, Margarida D. Amaral, Richard I. Morimoto

**Affiliations:** 1 Department of Molecular Biosciences, Rice Institute for Biomedical Research, Northwestern University, Evanston, Illinois, United States of America; 2 Faculty of Sciences, Centre for Biodiversity, Functional and Integrative Genomics (BioFIG), University of Lisboa, Lisboa, Portugal; 3 Department of Biomedical Engineering, Northwestern University, Evanston, Illinois, United States of America; 4 Centre of Human Genetics, National Institute of Health, Lisboa, Portugal; Brown University, United States of America

## Abstract

A hallmark of diseases of protein conformation and aging is the appearance of protein aggregates associated with cellular toxicity. We posit that the functional properties of the proteostasis network (PN) protect the proteome from misfolding and combat the proteotoxic events leading to cellular pathology. In this study, we have identified new components of the proteostasis network that can suppress aggregation and proteotoxicity, by performing RNA interference (RNAi) genetic screens for multiple unrelated conformationally challenged cytoplasmic proteins expressed in *Caenorhabditis elegans*. We identified 88 suppressors of polyglutamine (polyQ) aggregation, of which 63 modifiers also suppressed aggregation of mutant SOD1^G93A^. Of these, only 23 gene-modifiers suppressed aggregation and restored animal motility, revealing that aggregation and toxicity can be genetically uncoupled. Nine of these modifiers were shown to be effective in restoring the folding and function of multiple endogenous temperature-sensitive (TS) mutant proteins, of which five improved folding in a HSF-1–dependent manner, by inducing cytoplasmic chaperones. This triage screening strategy also identified a novel set of PN regulatory components that, by altering metabolic and RNA processing functions, establish alternate cellular environments not generally dependent on stress response activation and that are broadly protective against misfolded and aggregation-prone proteins.

## Author Summary

A common characteristic of protein conformational diseases is the appearance of protein aggregates associated with late-onset symptoms. Here, we have taken an unbiased genetic approach to test the hypothesis that protein aggregation and toxicity are co-linked genetic traits that are regulated by a common proteostasis network. To address this, we took advantage of the tractable genetic model *Caenorhabditis elegans* expressing expanded polyglutamines (polyQ) and performed a genome-wide RNA interference (RNAi) screen to identify genes that altered the proteostasis environment and suppressed aggregation and toxicity. These modifiers were subsequently tested on animals expressing mutant SOD1 and on animals expressing endogenous proteins with temperature-sensitive mutations. This screening triage resulted in the identification of nine genes with effects on protein folding, corresponding to new proteostasis pathways involved in metabolism and RNA processing functions.

## Introduction

Protein misfolding is an intrinsic aspect of protein biogenesis that, under optimal conditions, is kept in check by the properties of the proteostasis network (PN), and upon stress, aging, and expression of aggregation-prone proteins causes cellular dysfunction that places the organism at risk for diseases of protein conformation. These are common and prominent features in Alzheimer's disease (AD), Parkinson's disease (PD), Amyotrophic Lateral Sclerosis (ALS), polyglutamine disorders such as Huntington's disease (HD), muscular dystrophies, metabolic disorders, and certain cancers [1]–[5]. Each disease is associated with its own characteristic set of aggregation-prone proteins that differ in sequence, function and expression patterns. Nevertheless, protein misfolding and aggregation have similar consequences to the cell with deleterious consequences on gene expression, protein synthesis, folding, trafficking, clearance, and cell signaling.

The PN of molecular pathways coordinates protein synthesis, folding, trafficking and clearance [6]–[8] and determines the fate of proteins that do not acquire a native conformation. The chronic expression of aggregation-prone proteins, as occurs in conformational disorders, not only affects the function of proteins harboring mutations, but also challenges the stability of the PN, leading to the amplification of protein damage and persistent proteotoxicity [4], [9]–[12]. While protein aggregates and inclusions represent a prominent feature of many human diseases, it remains unanswered whether a cell responds identically to different protein aggregates. Elucidating the mechanism(s) by which misfolded proteins, oligomeric species, and/or aggregates interfere with cellular function represents a prominent challenge, given the complexity of the aggregation process, and the large number of cellular processes affected as proteostasis decline is propagated across tissues [6], [9], [13], [14]. Many studies have indicated that large inclusions correlate poorly with onset and severity of neurodegeneration and support a role for the soluble oligomeric species in toxicity [13]–[19]. Although intermediate species formed by distinct proteins have been suggested to display common structural motifs, it has been difficult to evaluate the contribution of different types of oligomers to toxicity [20]. Furthermore, despite the common theme of protein aggregation, growing evidence suggests that the cause of toxicity for each disease may, in part, be specific to the subset of molecular processes affected by the aggregated protein [21].

An alternative approach to understanding the origin of toxicity in each disease is to identify genetic modifiers that suppress aggregation and prevent the accumulation of metastable and misfolded proteins by enhancing global folding capacity [22]. Multiple *in vitro*, cell-based, and animal model systems have been developed to investigate the molecular events underlying aggregation-driven toxicity and identify modifiers of disease phenotypes [23]–[31]. While mammalian model systems are notoriously challenging to perform genome-wide screens due to the differences in genetic background and environment, screens performed in *Saccharomyces cerevisiae*, *Caenorhabditis elegans* and *Drosophila melanogaster* have identified processes that maintain proteome stability, promote folding and clearance. These include among others, molecular chaperones, proteasome subunits, components of the autophagy machinery, and the stress-induced transcriptional regulators DAF-16/FOXO and HSF-1 [32]–[39].

Here, we established a screening strategy in *C. elegans* to identify novel genetic modifiers of proteostasis that reshape the network to increase the cellular capacity for folding, prevent protein aggregation and suppress toxicity. Our approach was to identify components of the PN, that when down-regulated, enhance the functional properties of the PN to restore folding of the various folding sensors employed in the screen. This approach was designed to complement our previous efforts to identify enhancers of misfolding by screening for genes that when down-regulated caused the premature appearance of protein aggregates [35]. We identified 63 genetic modifiers that suppressed both polyQ and mutant SOD1 aggregation in muscle cells, of which 23 also suppressed associated toxicity. Of these, 9 modifiers systematically reduced the misfolding phenotypes of endogenous temperature-sensitive proteins. These modifiers were then characterized for dependence on HSF-1 activation and expression of cytosolic chaperones to enhance folding. This study introduces new proteostasis modifiers with a global effect on the stability of the muscle cell proteome, with likely broader relevance for conformational disorders.

## Results

### Screening Strategy for Genetic Modifiers of Protein Aggregation

We sought to determine whether modifier genes identified in a genome-wide RNAi screen for suppression of polyQ aggregation and toxicity in *C. elegans* would be efficacious on other disease-associated aggregation-prone proteins and endogenous metastable proteins. With this strategy, we tested the hypothesis of conserved modifier genes and pathways within the PN for protein misfolding and aggregation.

**Figure 1:**
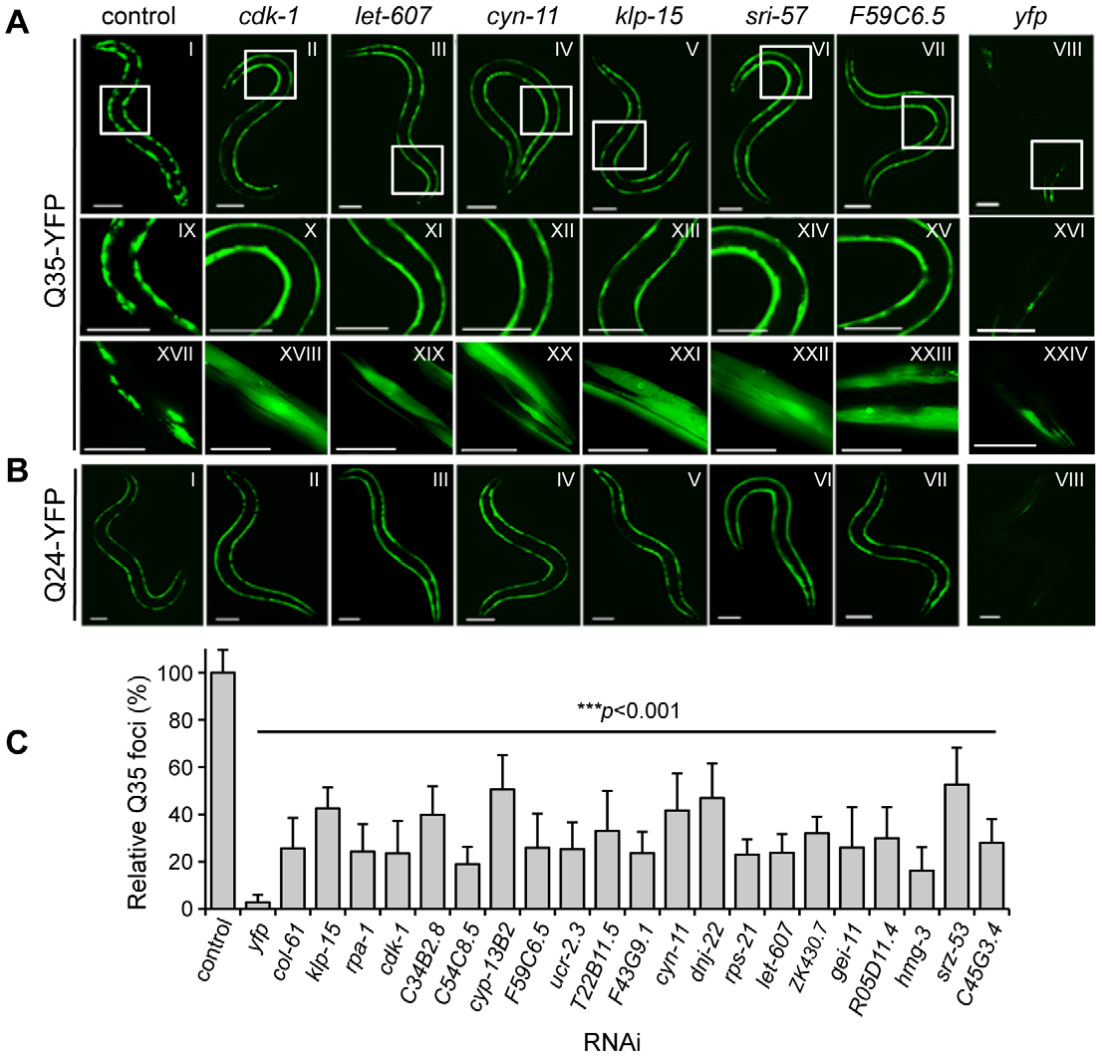
Genome-wide RNAi screen for suppression of Q35 aggregation. (A) Q35 animals (6 days old) show suppression of aggregation relative to EV control. Representative modifier genes: *cdk-1* (cyclin-dependent kinase); *let-607* (CREB/ATF transcription factor); *cyn-11* (cyclophilin); *klp-15* (kinesin-like protein); *sri-57* (serpentine receptor) and *F59C6.5* (NADH-ubiquinone oxidoreductase). *yfp*-RNAi is the control for RNAi efficiency. Panels IX–XXIV are higher magnification images of the boxed areas in I–VIII. Scale bars: 0.1 mm (I–XVI), 0.05 mm (XVII–XXIV)]. (B) Animals expressing soluble Q24 were used as a control for RNAi phenotypes dissociated from aggregation (scale bar 0.1 mm). (C) Q35 aggregate count (% foci relative to EV control) for a representative group of modifiers (±SD, *n*>3). Student t-test ***p<0.001 relative to control.

We initiated the screening strategy with a genome-wide RNAi screen to identify genes in *C. elegans* that, when knocked down, suppress aggregation of expanded polyQ::YFP fusion proteins expressed in body wall muscle (BWM) cells [26]. For this screen, animals expressing Q35 were used, as this is a threshold length for polyQ that exhibits adult onset protein aggregation and toxicity [26]. This screen was accomplished using a semi-robotic assay developed for feeding RNAi bacteria [40] to larval 1 (L1, day 1) stage Q35 animals [35]. During early development, Q35 protein is soluble in muscle cells until animals reach day 3 of age, when aggregation is first detected, and thereafter aggregation and toxicity increase with aging (Figure S1B, S1D) [26], [35]. Therefore, we selected day 6, corresponding to three days after the onset of Q35 aggregation (Figure S1B), to perform the RNAi screen to identify gene knockdowns that led to suppression of polyQ foci, relative to the empty vector (EV) control. RNAi against *yfp* was used as a control for the efficiency of RNAi gene-knockdown (Figure 1A: VIII, XVI, XXIV; Figure 1C). The screen was highly robust and identified 151 genetic modifiers that suppressed Q35 aggregation (Figure 1A, Table S1). Of these modifier genes, 91 exhibited a strong suppressor effect on aggregation by reducing the number of Q35 foci by 60 to 80% in ≥75% of the RNAi-treated animals (Table S1, Figure 1C). The remaining 60 modifiers gave more variable results and were less effective as suppressors (*i.e.* observed in ∼50% of the RNAi-treated animals with ∼50% reduction in foci; Table S1).

**Figure 2:**
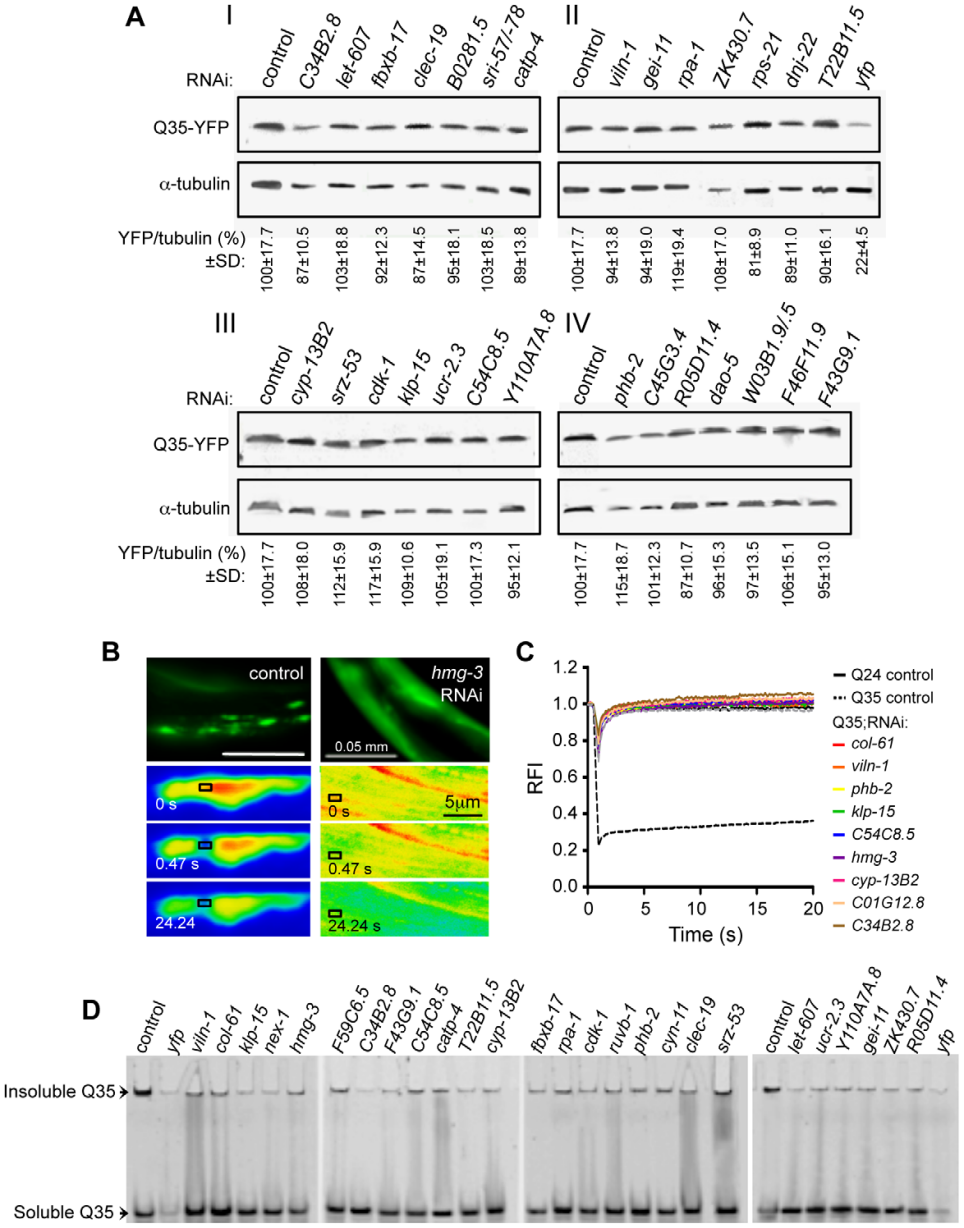
Suppressors of aggregation maintain polyQ in a diffuse state without affecting expression levels. (A) SDS-PAGE and western blotting analysis of protein samples from Q35 RNAi-treated animals (6 days old), immunoblotted with anti-YFP (32 KDa) and anti-α-tubulin (55 KDa) antibodies. Control refers to EV RNAi (I–IV). YFP/tubulin ratios were calculated from protein band intensities and are shown as an average % of the control (±SD, from ≥3 biological replicates, Student t-test *p*>0.05). *yfp*-RNAi in panel II is the positive control for reduced protein levels. (B,C) FRAP analysis confirms suppression of polyQ aggregation to a diffuse state. (B) Q35 protein was subjected to photobleaching in animals treated with control RNAi (left) or *hmg-3* RNAi (right) and fluorescence recovery was measured at the indicated time points. (C) Quantitative FRAP analysis indicates the relative fluorescence intensity (RFI) at each time point, and it represents an average of ≥12 independent measurements for each RNAi (5 for the controls). The soluble Q24 control is shown in black solid line, and the Q35 foci control in black dashed line. (D) Native PAGE analysis of whole protein extracts from 6 day old Q35 animals treated with RNAi. Q35 aggregated protein retained at the top of the gel was reduced by each of the modifiers tested.

We next used a counter-screen with animals expressing soluble Q24, as these animals do not exhibit aggregation or toxicity [26], to control for phenotypic changes caused by RNAi that are not related to aggregation, such as changes in YFP fluorescence, body morphology and size, egg-laying and sterility (Table S1). We observed that none of the Q35 aggregation modifiers had any effect on Q24 protein (Figure 1B), suggesting that the RNAi suppressor effect was not due to transgene silencing. The modifiers that did not meet these criteria or had deleterious consequences on animal development and viability were not studied further. Moreover, to assess whether suppression of aggregation was due to changes in polyQ expression, we examined mRNA and steady-state protein levels for a representative group of modifiers. We quantified the levels of *q35::yfp* mRNA by rt-PCR (Figure S2) and the levels of Q35::YFP protein by SDS-PAGE and western blot analysis (Figure 2A). The results show that, for the RNAi modifiers tested, suppression of aggregation occurred without affecting the polyQ mRNA or protein levels.

To obtain evidence that the suppression of visible Q35 aggregates corresponds to the appearance of soluble Q35 protein (Figure 1A), we used the dynamic imaging method of Fluorescence Recovery After Photobleaching (FRAP). Inclusion-localized Q35 corresponds to an immobile state with very limited fluorescence recovery following photobleaching (Figure 2B and 2C: Q35 control) [23], [35], whereas the fluorescence of diffuse-looking Q35 in animals fed with modifier RNAi recovered immediately, consistent with a diffuse and soluble state analogous to soluble Q24 (Figure 2B *hmg-3* and Figure 2C). These results provide biophysical evidence for Q35 solubility identified by the visual screening. We further examined the biochemical properties of Q35 in total protein extracts, for a representative group of modifiers. We found that the amount of aggregated polyQ protein detected using native PAGE analysis was reduced, and that the levels of soluble and diffuse species were increased (Figure 2D). Taken together, these results reveal that RNAi knockdown of specific modifier genes suppressed polyQ aggregation by maintaining the protein in a mobile soluble state.

The identity of the RNAi-targeted genes was verified by sequencing of the dsRNA plasmids, followed by Blast analysis in NCBI and Wormbase databases. The Q35 aggregation modifier genes are 88% conserved, with predicted human orthologs, and can be grouped into seven functional categories of cell cycle, DNA synthesis and repair; RNA synthesis and processing; protein synthesis; protein folding and turnover; cell structure and protein trafficking; signaling; and energy and metabolism (Table S1, Figure S3A, S3B). The fraction of modifiers represented in each functional class is significantly distinct from their representation in the *C. elegans* RNAi library (Figure S3A, S3B) [41], indicating enrichment for cellular processes important for proteostasis.

**Figure 3:**
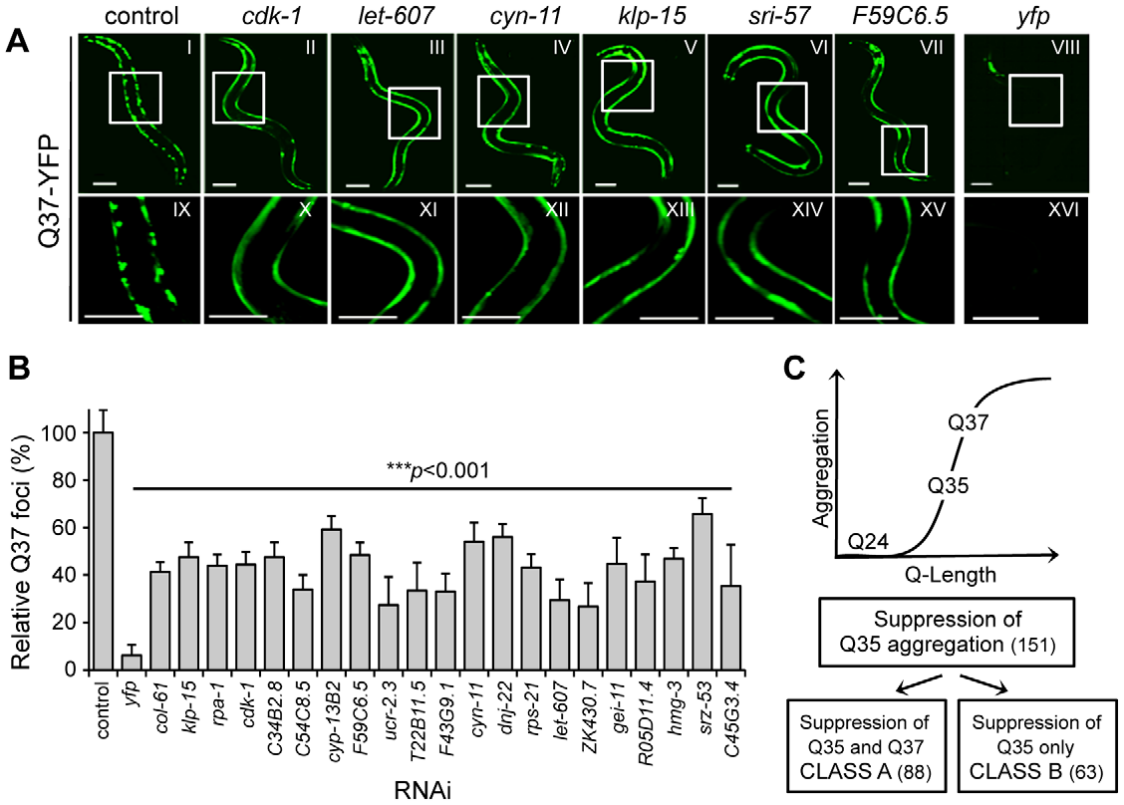
Common RNAi suppressors of Q35 and Q37 aggregation. (A) Counter-screen in 5 day old Q37 animals to identify the strongest suppressors of polyQ aggregation. Panels IX–XVI show a higher magnification image of the boxed areas on I–VIII. Scale bar is 0.1 mm. (B) Q37 aggregate count (% foci relative to EV control) for a representative group of modifiers (±SD, *n*>3). Student t-test ***p<0.001 relative to control. (C) Screen strategy: genome-wide screen with the threshold Q-length for aggregation Q35, and counter-screens with the soluble Q24 and the higher Q-length Q37 strains. Class A refers to Q35 and Q37 common strong modifiers, and Class B to modifiers only affecting Q35.

We next asked whether the Q35 modifiers were effective on another polyQ model as a way to distinguish the most robust polyQ aggregation suppressors. This was done by screening a transgenic line expressing Q37::YFP. These animals exhibit a more rapid onset of aggregation relative to Q35 animals, between day 2 and 3 of age, together with a more rapid decline in motility (Figure S1A, S1B, S1D). These phenotypes are dependent solely upon the CAG-repeat length as the levels of Q35 and Q37 are identical (Figure S1C). Q37 animals were fed RNAi from L1 stage (day 1) and aggregation was examined on day 5, corresponding to three days post-aggregation onset (Figure 3A, 3B). Of the initial 151 modifiers of Q35 aggregation, only 88 of these also suppressed Q37 aggregation, of which 81 corresponded to the strongest Q35 suppressors (Table S1). We designated the set of common modifiers of Q35 and Q37 aggregation as Class A strong modifiers (Table 1), and the remaining 63 genes as Class B weak modifiers (Figure 3C).

**Table 1:** Overview of the modifiers that suppress Q35 and Q37 (Class A) aggregation in *C. elegans* BWM cells.

Process(# Genes)	Molecular Function (# Genes)	Gene
**Cell Cycle DNA Synthesis and Replication (8)**	Cell Cycle (1)	*cdk-1*
	DNA recombination (2)	*fbxb-11; fbxb-17*
	Meiosis (1)	*F46F11.9*
	Replication (4)	*mcm-2; rnr-2; rpa-1; ruvb-1*
**Cell structure and Protein transport (6)**	Cellular matrix, cuticle (4)	*ppn-1; col-61; col-69; viln-1*
	Vesicle trafficking (2)	*klp-15; nex-1*
**Energy and Metabolism (17)**	Metabolism (9)	*C50D2.2; R03D7.1; C47F8.4; C54C8.5; D2030.1; elo-2; W06H3.3; F43G9.1; T22B11.5*
	Electron transport chain (5)	*C34B2.8; F59C6.5; ucr-2.3; cyp-13B2; cyp-33D1*
	Ion transport (2)	*B0281.5; catp-4*
	Mitochondrial function (1)	*F43E2.7*
**Protein Folding and transport (7)**	Chaperone (6)	*F08H9.3; cyn-11; cyn-12; C30C11.4; dnj-22; phb-2*
	Protein glycosylation (1)	*tag-335*
**Protein Synthesis (10)**	Mitochondrial ribosome (3)	*C26E6.6; F33D4.5; mrpl-41*
	Ribosomal protein (3)	*rpl-2; rpl-35; rps-21*
	Translation (4)	*krs-1; F17C11.9; H19N07.1; R05D11.4*
**RNA Synthesis and Processing (13)**	Nucleosome binding (2)	*hmg-3; hmg-4*
	Ribosome biogenesis (1)	*C15H11.9*
	RNA processing, splicing (5)	*F13H8.2; fib-1; let-716; ZK430.7; Y110A7A.8*
	Transcription (5)	*C55A6.9; K03F8.1; fkh-6; gei-11; let-607*
**Signaling (10)**	Receptor protein (6)	*dao-5; xpo-1; sri-57; sri-78; srz-53; T11F1.6*
	Transduction (4)	*clec-19; pdl-1; rgl-1; W03B1.9*
**Unknown (17)**	Unknown (17)	*C23G10.10; C45G3.4; F14B8.2; F26E4.2; F47F2.3; K02E7.11; ptr-19; R05D7.2; ril-1; smu-1; T04D3.5; vab-19; F53F10.1; W03B1.5; Y51H7BR.3; fbxa-76; F54C4.3*

### Identification of Common Modifiers of PolyQ and Mutant SOD1 Aggregation

**Figure 4:**
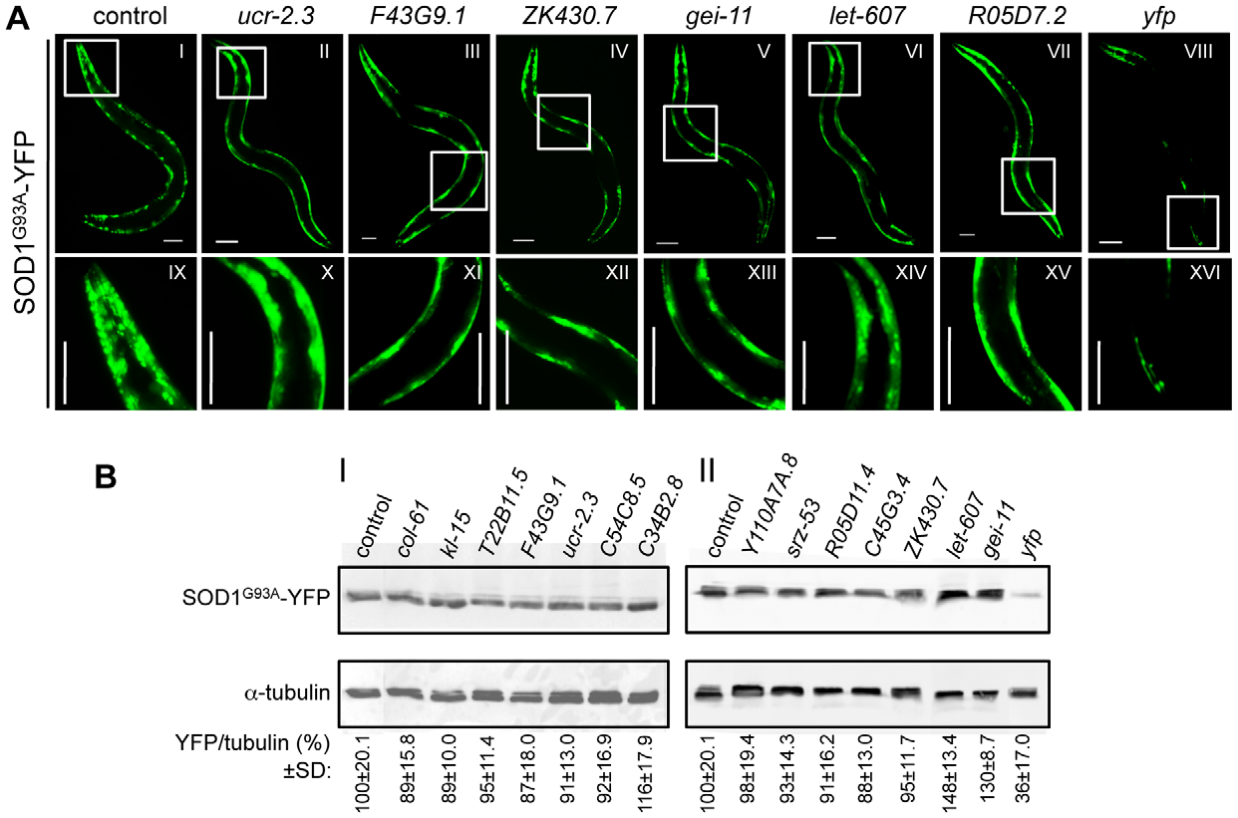
PolyQ aggregation modifiers tested in the mutant SOD1^G93A^ model. (A) Representative RNAi suppressors of SOD1^G93A^ aggregation: *ucr-2.3* (ubiquinol cytochrome c reductase); *F43G9.1* (isocitrate dehydrogenase); *ZK430.7* (sof1-like rRNA processing protein); *gei-11* (GEX-3-interacting protein, Myb transcription factor); *let-607* (CREB/ATF transcription factor); *R05D7.2* (unknown) and *yfp* RNAi control. Higher magnification images (IX–XVI) of the boxed areas (I–VIII) show reduced number of SOD1^G93A^ foci in animals treated with RNAi, relative to the EV control. Scale bar is 0.1 mm. (B) SDS-PAGE and western blot analysis of protein samples from SOD1^G93A^ RNAi-treated animals (5 days old), immunoblotted with anti-YFP (32 KDa) and anti-α-tubulin (55 KDa) antibodies. Control refers to EV RNAi. YFP/tubulin ratios (bottom) were calculated from protein band intensities and are shown as an average % of the control (I, II) ±SD, from ≥3 biological replicates (Student t-test *p*>0.05). *yfp*-RNAi (II) is the positive control for reduced protein levels.

We next tested whether the genetic modifiers of polyQ aggregation would be effective on yet another model that expresses the mutant human SOD1^G93A^. This model shows aggregation onset during embryonic development and a distinctive pattern of foci that persists throughout adulthood (Figure S1E, S1F) [12]. Of the 88 polyQ aggregation modifiers, 63 also suppressed mutant SOD1^G93A^ aggregation in 5 day old animals (Figure 4A, Table S1), without causing phenotypic changes in SOD1^wt^ animals (not shown). A subset of these common aggregation suppressors were also examined by SDS-PAGE and western blot analysis, and shown not to reduce steady-state protein levels of SOD1^G93A^ (Figure 4B). 95% of the mutant SOD^G93A^ suppressors belong to the polyQ Class A modifiers (Table S1). Unlike Q35 aggregation that is only detected in young adult animals, mutant SOD^G93A^ aggregation occurs in embryos [12], and yet many Class A modifiers were effective suppressors of SOD1 aggregation providing additional support that Class A modifiers are robust modifiers of protein folding. Moreover, these modifiers, that are common to polyQ and SOD1, exhibit a similar overall distribution into functional classes (Figure S3C) as was described for modifiers of polyQ aggregation (Figure S3B), thus identifying new modifier pathways that are common to protein aggregation.

We propose that these new modifiers can either suppress aggregation directly by affecting cellular processes that mediate aggregate formation, or indirectly by altering some aspect of the PN that confers a protective action that increases folding. To distinguish between these two possibilities, we employed genetic tests to determine which modifiers reflect an improvement of the folding environment, by reducing misfolding and associated toxicity.

### Suppression of Aggregation Can Be Uncoupled from Toxicity

Protein aggregation is a common feature of many diseases; however, the relationship between aggregation and cellular toxicity remains controversial. The appearance of aggregates and inclusions has been linked both to cellular dysgenesis and toxicity, as well as protection from toxicity [13]–[19]. Therefore, we took advantage of an unbiased genetic approach to test the relationship between suppression of aggregation and toxicity. Because the initial genetic screens were based solely on aggregation phenotypes, we were able to subsequently perform cellular toxicity assays to assess this relationship.

**Figure 5:**
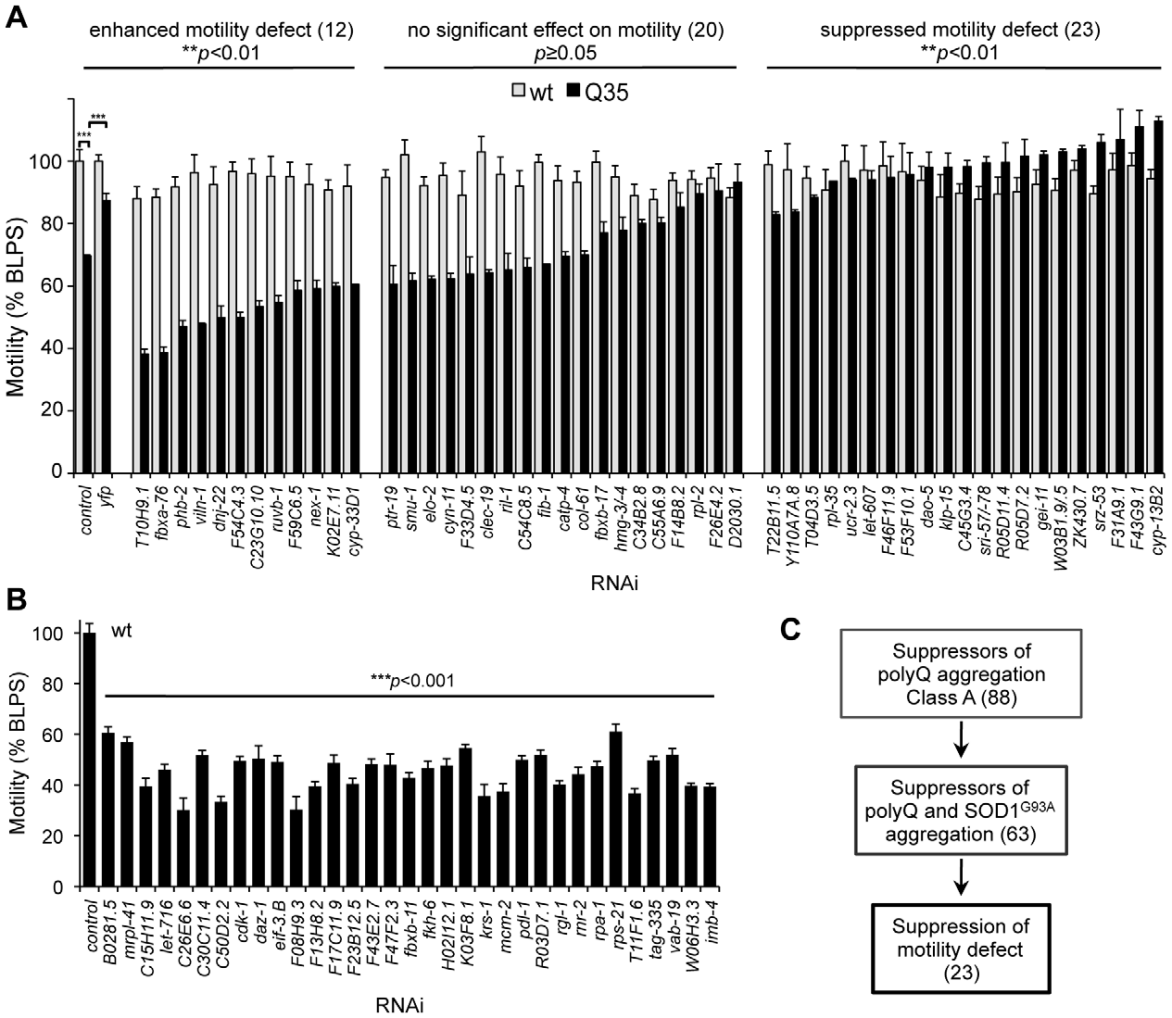
Suppression of polyQ aggregation and toxicity are genetically uncoupled. (A) Motility measurements of Q35 (black) and wt (grey) animals treated with aggregation suppressor RNAi. Motility is measured in body-length per second relative to wt motility in EV control (% BLPS±SEM). Shown here are the modifiers that did not affect wt motility, grouped in three classes that: enhance Q35 motility defect (Student t-test **p<0.001); cause no change (Student t-test *p*≥0.05); or suppress Q35 motility defect (Student t-test **p<0.01) relative to Q35 control. Statistical significance between classes calculated by 1-way ANOVA ***p<0.0001; and t-test ***p<0.001. (B) Aggregation modifiers that caused a deleterious effect on wt motility (% BLPS±SEM, relative to EV control). Student t-test ***p<0.001 relative to control. (C) Screening triage for modifier suppressors of protein aggregation and toxicity.

Relative to wt animals or animals expressing soluble polyQ (Q24), Q35 animals exhibit muscle dysfunction resulting in a 40% loss of motility at 6 days of age (Figure S1D) [26]. Therefore, we quantified the motility of RNAi-treated Q35 animals as a measure of polyQ-associated cellular toxicity, using an automated worm tracker system analysis, validated by manual methods. As a reference positive control for toxicity suppression, we show that motility was restored to near wt levels by knockdown of the Q35 transgene expression with *yfp*-RNAi (Figure 5A). All 88 Class A modifiers were tested for effects on the motility of Q35 animals and wt control animals (Table S2). Because we sought to identify improvement of motility directly associated to suppression of Q35 aggregation, we excluded any modifier that, alone, had effects on the motility of wt animals. Of the 88 modifiers tested in wt animals, 33 gene knockdowns (37%) affected the motility of wt animals (Figure 5B), and were excluded from further analysis. For the remaining 55 modifiers, 42% improved the motility of Q35 animals to wt levels, 36% had no effect, and 22% enhanced the toxicity of Q35 (Figure 5A). These results revealed that suppression of aggregation, as detected by visual, biophysical, and biochemical measures, does not necessarily predict that the physiological health of the cell will be restored. The genetic uncoupling between aggregation and toxicity further reinforces previous similar observations [13], [14], [19]. Taken together, the toxicity in diseases of protein conformation is the outcome of a complex series of misfolding events, involving multiple species and aberrant interactions within the cell.

### Identification of a Core PN by Screening with Endogenous Metastable Proteins

Among the challenges with studies of protein misfolding and aggregation have been the concerns with the physiological imbalance associated with overexpression of heterologous proteins in the respective model systems. To circumvent this concern, we asked whether the PN modifiers that suppressed polyQ and mutant SOD1 aggregation (Figure 5C) would also restore the folding of endogenous metastable proteins harboring temperature sensitive (TS) mutations. TS-mutations represent an important class of highly sensitive folding sensors that are expressed at normal endogenous levels and have quantifiable phenotypes when properly folded at the permissive condition or misfolded at the restrictive temperature [10], [42], [43]. This strategy was also used to distinguish between modifiers that directly perturb and suppress the formation of protein aggregates, from the modifiers that reshape the PN to improve the protein-folding environment.

**Figure 6:**
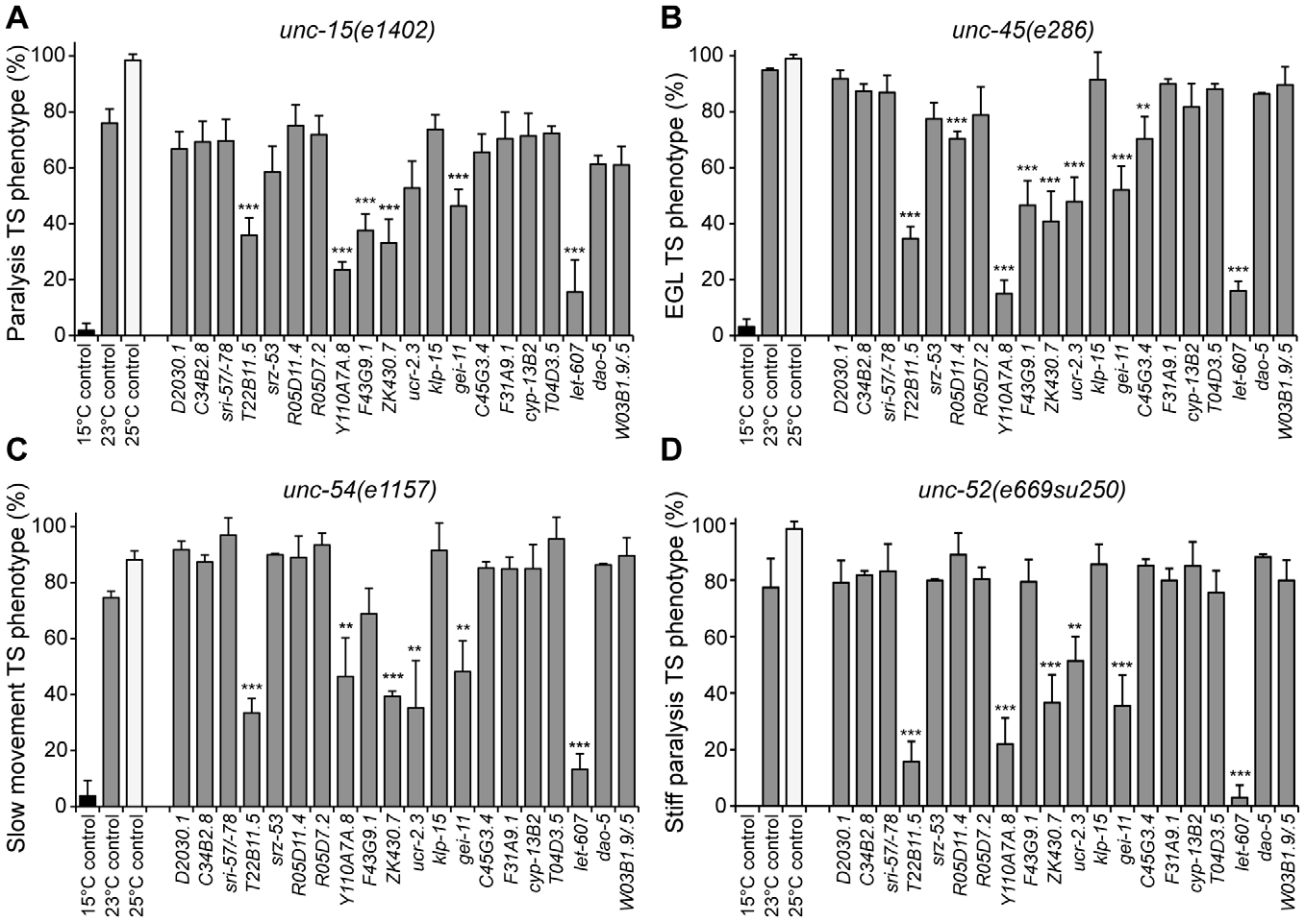
Aggregation modifiers that rescue the folding of endogenous TS mutant proteins. Modifiers of aggregation and toxicity were tested on endogenous muscle TS mutant proteins. 15°C is the permissive temperature, 25°C is the restrictive temperature and 23°C is the temperature used for RNAi. Misfolding of TS mutant proteins was assessed by measuring the % of animals displaying the associated muscle dysfunction phenotype: (A) *unc-15(e1402)* (paramyosin), uncoordinated/slow movement; (B) *unc-45(e286)* (myosin assembly protein), egg laying and paralysis defect; (C) *unc-54(e1157)* (myosin), slow movement/paralysis; (D) *unc-52(e669su250)* (perlecan), stiff paralysis (±SD, *n*>3, Student t-test relative to 23°C control **p<0.01, ***p<0.001). Statistical comparison using 1-way ANOVA ***p<0.001.

**Figure 7:**
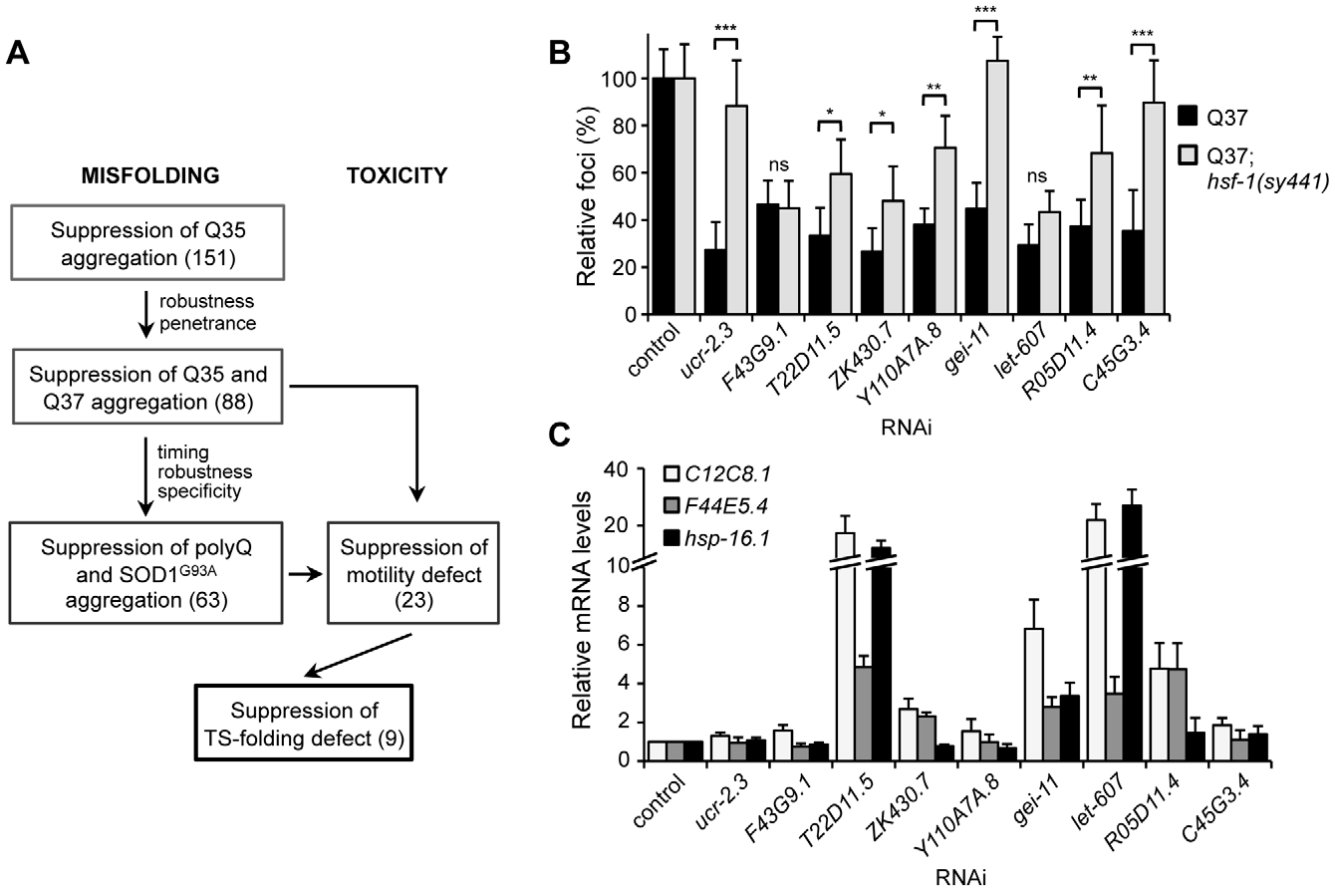
Core PN modifiers and activation of the heat shock response. (A) Screening strategy to identify genetic modifiers that enhance the folding environment and are effective in multiple misfolding models. (B) Suppression of polyQ aggregation by the final nine modifiers dependence on HSF-1. Aggregate quantification on RNAi-treated Q37;*hsf-1(sy441)* hypomorphic mutant animals relative to control (±SD). Student t-test *p<0.05; **p<0.01; ***p<0.001 (ns, non-significant). (C) Real-time qPCR analysis of the levels of *hsp* (*C12C8.1, F44E5.4, hsp-16.1*) genes in RNAi-treated wt animals. Data are relative to the levels of each gene in wt;EV control (±SD) from 3 biological replicates.

We examined the properties of four TS mutant proteins corresponding to the paramyosin ortholog UNC-15, the basement-membrane protein perlecan UNC-52, the myosin-assembly protein UNC-45, and the myosin heavy chain UNC-54 [10]. At the permissive temperature (15°C), each of these TS-proteins is known to be fully functional and animals harboring these mutants exhibit a wt phenotype, whereas at the restrictive temperature (23°C or 25°C) these TS-proteins misfold and cause muscle dysfunction that can be measured as slow movement and paralysis (UNC-15, UNC-54, Figure 6A, 6C), egg-laying defects leading to swelling and paralysis (UNC-45, Figure 6B), and stiff-paralysis (UNC-52, Figure 6D) (see Materials and Methods) [10], [42], [44]. These phenotypes are specific to animals expressing the TS mutations, and are not observed in wt animals. We tested all 23 RNAi modifiers that suppressed both Q35 aggregation and toxicity (Figure 5C), on each of the TS strains, and found that a total of nine modifiers reduced the number of animals displaying TS phenotypes by 40% to 90% at 23°C, a slightly lower restrictive temperature at which the efficiency of the RNAi protocol was maintained (Figure 6A–6D, Table 2). These results suggest that while protein misfolding is common to all three classes of folding sensors (polyQ, mutant SOD1, and TS-mutant proteins), the cellular environment and the PN are influenced differentially by the modifiers, as reflected by the effects on these sensors. We propose that these final nine modifiers (Figure 7A) are core PN modulators that confer improvement of cellular folding capacity in *C. elegans* muscle cells.

**Table 2:** Core modulators of protein homeostasis in *C. elegans* BWM cells.

Gene/Cosmid	Class	Molecular function	Human ortholog	Expression	TS	Direct Interactors (STRING 9.0)
*ucr-2.3*	EM	Ubiquinol cytochrome c reductase, complex III ETC	UQCRC2, Cytochrome b-c1 complex III	Ubiquitous, mitochondrial	3/4	ETC complex III subunits: *cyc-1, isp-1, ucr-2.2, T27E9.2, F45H10.2, T02H6.11, R07E4.3*. *F57B10.14* (unknown).
*F43G9.1*	EM	Isocitrate dehydrogenase α-subunit, TCA cycle	IDH3A, Isocitrate dehydrogenase, α-subunit	Ubiquitous, mitochondrial	2/4	Aconitase (*aco-1, aco-2*), ATP synthase (*atp-2*), Isocitrate dehydrogenase subunits (*C37E2.1, idh-1, idh-2, C30F12.7, F35G12.2)* OGDH subunits (*T22B11.5, ZK836.2*)
*T22B11.5*	EM	2-oxoglutarate dehydrogenase, E1 subunit, TCA cycle	OGDHL, 2-oxoglutarate dehydrogenase E1	Ubiquitous, mitochondrial	4/4	OGDH subunit (*W02F12.5, ZK836.2*), Isocitrate dehydrogenase subunits(*idh-1, idh-2, C37E2.1,C30F12.7, F43G9.1*), LLC1.3, glutaryl-CoA-dehydrogenase (*F54D5.7),* Malonyl-CoA decarboxylase (*F35G12.1)*
*ZK430.7*	RSP	Sof1-like rRNA processing protein	WDSOF1, WD repeat and SOF domain-containing protein 1	Muscle enriched, nucleus	4/4	rRNA processing (*fib-1, C05C8.2*), ribosome (*C48B6.2, nep-1, F13H8.2*), acyltransferase (*F55A12.8)*, tryptophan protein 2 (*F55F8.3*), Bystin (*byn-1*), nucleolar protein (*nol-10*)
*Y110A7A.8*	RSP	Putative mRNA splicing factor PRP31	PRPF31, U4/U6-SNRP	Oogenesis enriched	4/4	RNA processing and splicing: *prp-8, M28.5, Y59A8B.6, prp-4, rpl-3, ism-6. M03C11.4* (unknown)
*gei-11*	RSP	GEX-interacting protein 11 MYB-family	SNAPC4, MYBL2, MYB, MYBL1	Nucleus	4/4	Acetylcholine receptor subunit (*unc-29*), tissue morphogenesis (*gex-3*)
*let-607*	RSP	CREB-ATF transcription factor	CREB3L3/L4, CREBH transcription factor	BWM, neurons, vulva, pharynx, spermatheca, ER, nucleus	4/4	-
*R05D11.4*	PS	ATP-dependent RNA helicase, translation	DDX52, ATP-dependent RNA helicase	Intestine, muscle, cytoplasm	1/4	-
*C45G3.4*	U	Unknown	Unknown	-	1/4	-

**Key:** EM (energy and metabolism); RSP (RNA synthesis and processing); PS (protein synthesis); U (unknown); TS (temperature sensitive mutant proteins showing suppression of phenotype/total tested).

### Folding Enhancement by Activation of the Heat Shock Response

Upstream of the core components of the PN is the master cytosolic stress-responsive pathway that leads to HSF-1 activation and expression of molecular chaperones for stability of the proteome. To examine whether the nine PN modifiers (Figure 7A) lead to HSF-1 activation as a general mechanism for proteostasis improvement, we introduced a hypomorphic mutation of *hsf-1(sy441)* into the background of the polyQ strain Q37 and knocked-down each modifier gene (Figure 7B). Our results show that: suppression of aggregation by *ucr-2.3*, *gei-11* and *C45G3.4* was completely dependent on HSF-1; whereas *T22D11.5*, *ZK430.7*, *Y110A7A.8* and *R05D11.4* exhibited a weaker dependence on HSF-1; and *F43G9.1* and *let-607* were independent of HSF-1. We next examined whether chaperone gene expression was affected downstream of the nine PN modifiers, by monitoring the expression of Hsp70 (*C12C8.1, F44E5.4*) and small Hsp (*hsp-16.1*), upon knockdown of each genetic modifier (Figure 7C). We show that five of nine PN modifiers induced expression of cytoplasmic chaperones. Knockdown of *let-607* (ER-UPR, Table 2) had the strongest effect, suggesting an important regulatory crosstalk between cytoplasmic and ER stress response pathways. Knockdown of the TCA cycle component *T22B11.5* led to upregulation of chaperones and establishes a link between folding and metabolic state, whereas knockdown of *gei-11* (putative negative regulator of cholinergic signal, Table 2) induction of *hsp* suggests an effect of cholinergic signaling on muscle homeostasis (Figure 7C). Reduction of *R05D11.4* (translation) leading to induction of *hsp-70* was consistent with an effect of protein synthesis on folding machinery. For the remaining four PN modifiers, knockdown of *ZK430.7* (RNA processing) had a modest effect on *hsp* expression, while knockdown of *Y110A7A.8* (splicing), *ucr-2.3* (ETC) and *F43G9.1* (TCA cycle) had no effects on *hsp* levels (Figure 7C, Table 2). This suggests that proteostasis was restored through other pathways involving reduced metabolism and energy production. Taken together, these results demonstrate that improvement of the cellular folding environment by these novel proteostasis modulators is not simply a consequence of a generalized induction of the heat shock response and molecular chaperones but also involves other PN pathways, not previously linked to proteome surveillance.

## Discussion

Genetic screens have provided invaluable insights into biological processes, and have the advantage of being unbiased and entirely based on endpoint phenotypes. We have employed a genetic strategy to explore how the proteostasis network can be genetically modulated to increase cellular protection against the toxicity of protein aggregation (Figure 7A). This strategy benefitted from certain advantages of *C. elegans* as a metazoan model for the expression of conformationally challenged proteins and the ability to screen the genome for modifiers that suppress both aggregation and toxicity by gene knockdown. This complements the candidate gene approaches that led to the identification of molecular chaperones, ubiquitin proteasome, autophagy, and the upstream stress response signaling pathways. The use of a screening triage approach (Figure 7A) revealed that genetic modifiers that suppress aggregation of polyQ are also effective with mutant SOD1, and revealed the identity of candidates of a core PN. However, only a subset of these suppressors of aggregation also suppressed cellular toxicity, a result that is consistent with the genetic uncoupling between aggregation and toxicity observed in various disease models [13]–[19]. This screen ultimately provided a filter that led to identification of a final set of nine core muscle PN modifier genes (Table 2), not previously shown to be directly involved in protein homeostasis and the restoration of folding in both animal models of protein aggregation and endogenous metastable proteins.

### New Genetic Modulators of Protein Aggregation

The genome-wide screen for suppression of polyQ aggregation identified a collection of modifier genes from distinct cellular functional classes (Table S1, Figure S3B). Modifiers in the category *cell structure and protein transport*, include cytoskeleton components (filamin) and matrix proteins (*ppn-1, mua-3, gon-1*), supporting observations that aggregation can be affected by disturbing the integrity of cell structure [10]. Other genes in this group encode motor proteins involved in vesicular trafficking (*klp-15, nex-1*), consistent with a role for protein movement and transport in aggregation. *Cell growth and replication* modifiers are involved in progression through the cell cycle (cyclin-dependent kinases), and DNA replication and recombination (transposases), with a likely general effect on growth rate and development. *Energy and metabolism* modifier genes are involved in energy production and mitochondrial electron transport chain (ETC) function. Restriction in energy levels not only affects overall protein biogenesis, as it is a highly ATP-dependent process, but also, metabolic enzymes can influence protein folding in the cell by altering the levels of organic/inorganic solutes with effects in polypeptide chain solvation [6]. Notably, reduced ATP synthesis, ETC activity or mitochondrial function have been shown to enhance lifespan, possibly by delaying age-dependent decline in protein folding capacity and by upregulation of stress-response pathways that promote proteostasis and survival [45]–[48]. *Gene expression and protein synthesis* related modifiers function in RNA metabolism, ribosome biogenesis, and protein synthesis. This is consistent with reduced translation increasing *C. elegans* lifespan, perhaps by activating a physiological state with increased stress resistance and folding capacities [49]–[51]. *Post-translational control* modifier genes are involved in chaperone-assisted folding, such as HSP70 superfamily members, DNAJ co-chaperones and cyclophilins, and post-translation modifying enzymes such as SUMO and E3-ubiquitin ligases. The role of chaperones on protein solubility, misfolding and aggregation has been well established [52], [53], and an imbalance in certain co-chaperones has been suggested to alter chaperone activity in the cell and folding [54]. *Signaling* RNAi-targeted genes included nuclear hormone receptors, G-protein-coupled receptors, C-type lectins (endocytic receptors), or calcium export and channel-transport activity. These regulators affect reproduction, growth, morphogenesis (development), and locomotion by altering signaling pathways involved in neuronal and muscle function.

### Genetic Screens for Enhancer and Suppressor Modifiers of PolyQ Aggregation

Information retrieved from comparative analysis of genetic screens of different misfolded proteins can provide important insights to identify both common and protein-specific pathways for conformational disorders (Table S3). Highly relevant to this point is our ability to compare the modifier genes identified in this study with a previous complementary screen using the same threshold Q-length properties of the Q35 model to identify genes that when knocked-down by RNAi led to premature onset of polyQ aggregation [35]. Together, these genome-wide screens identified 341 genetic modifiers that cluster into the same functional classes and pathways, but correspond to distinct genes within these pathways. Specific modifier genes may interfere with the misfolded species at different stages of the aggregation process with opposite outcomes, consistent with the functional properties of a network, where different components within a process can shift the equilibrium in opposing directions to alter the stability of the proteome, to intensify or suppress the polyQ phenotype.

While it might seem counter-intuitive that molecular chaperones could suppress polyQ aggregation when knocked-down, this is consistent with observations that proteome stability can be enhanced or suppressed by changing the composition of the cellular chaperome [52]–[55]. For example, reducing the expression of *cyn-11* and *cyn-12* (cyclophilin D isoforms) and *dnj-5*, that function primarily as co-chaperones to regulate Hsp70 and Hsp90 activities [56], promotes a polyQ soluble state. These co-chaperones could function as negative regulators of chaperone function, and their down-regulation results in enhanced chaperone activities leading to suppression of misfolding [52], [57], [58]. This would be consistent with evidence that Hsp70 folding activity is negatively regulated by co-chaperones and co-factors such as CHIP [59] and BAG-1 [60]. Therefore, enhancement of folding can be achieved by both positive and negative regulation of chaperones or by a compensatory response that up-regulates other chaperones.

Another functional class common to both Q35 screens is the protein trafficking and cell matrix. Suppression of aggregation by knockdown of cell cytoskeleton proteins, such as intermediate filaments (MUA-3 and MUA-6) and filamin, is supported by experimental evidence that the dynamics of aggregation rely in part on translocation of proteins into inclusions. For example, the active transport of Htt-exon1 along microtubules has been shown to be required for inclusion body formation [61]. In contrast, premature aggregation was observed when the expression of vesicle proteins involved in protein trafficking was knocked-down [35], including TFG-1 COP-II complex, APT-3 and APT-1, and cell membrane assembly proteins (SNAP-25). Interference with these processes can disturb essential steps of the folding and secretory pathways, increase the load of misfolded proteins in the cell and lead to premature polyQ aggregation.

### Suppression of PolyQ Aggregation and Toxicity Can Be Uncoupled

An important observation from these studies is that the Class A PN modifiers (Figure 3C, Table 1) were highly effective to suppress polyQ aggregation, and yet only 42% of these modifiers also reduced toxicity, with approximately equal numbers of modifier genes with either no effect on toxicity or even enhancing toxicity (Figure 5A). These results provide independent evidence that suppression of aggregation alone does not predict that the physiological health of the cell will be restored. From a mechanistic perspective, it is increasingly clear that a series of events are associated with the conversion of the nascent polyQ protein into different oligomeric states, immobile aggregate species and inclusion bodies [62]–[65]. We conclude that the genetic suppression of aggregation can occur via a wide range of mechanisms that are dissociated from the effect on toxicity, consistent with previous observations that interference with the aggregation process could in some cases enhance the formation of “toxic oligomeric species” [13]–[19], [66]–[68]. Moreover, each modifier gene is certain to function within its own network of interacting partners (Table 2), revealing an expanding network through which each modifier can suppress aggregation, but with differential effects on toxicity depending on the affected cellular function. The demonstration that protein aggregates are uncoupled from cellular toxicity has implications for the understanding of the PN and for development of therapeutics.

### Triage Screening Method for Identification of Core Modulators of BWM Proteostasis

We took advantage of *C. elegans* models of protein aggregation-toxicity in addition to folding sensor strains harboring TS mutations in endogenous proteins to identify a core group of modulators that improve folding in muscle cells (Figure 7A). Our results highlight important aspects of the PN, relevant for both ‘gain-of-toxic function’ by aggregation-prone proteins, and ‘loss-of-function’-derived toxicity due to protein misfolding. The nine PN modifiers that remained at the end of the screening tree function in the mitochondrial respiratory chain and TCA cycle, that regulate metabolism, energy balance and prevention of oxidative stress, in addition to rRNA processing and transcription, that determine gene expression and proteome load (Table 2). While these modifier genes, upon initial inspection, seem not to be directly involved in folding, perturbation of their specific functions and networks of interactions re-adjusts the PN to enhance its capacity, as suggested by activation of the heat shock response and chaperone expression (Figure 7B, 7C). Moreover, comparison to other genetic screens performed in *Drosophila* [32], [33], [39], *C. elegans* [34]– and yeast [38], [71] (Table S3) provides additional insights into the regulation of the PN by these modifiers. *ucr-2.3* encodes an ubiquinol-cytochrome c reductase subunit of the mitochondrial respiratory chain, and *F43G9.1* and *T22B11.5* encode TCA-cycle enzymes (isocitrate dehydrogenase and 2-oxoglutarate dehydrogenase, respectively) (Table 2). Disruption of the respiratory chain and energy production has been suggested to have consequences on cellular homeostasis [47], [48]. Intriguingly, knockdown of *ucr-2.3* was shown to enhance the toxicity of human Tau expressed in *C. elegans* neuronal cells (Table S3) [34]. This discrepancy may be related to Tau-specific proteotoxicity, not derived from aggregation or misfolding, but associated with microtubule binding and disruption. *F43G9.1* was also identified as a enhancer of lifespan [70], which is consistent with a role in proteostasis. *ZK430.7* encodes an rRNA processing factor, *Y110A7A.8* is a putative mRNA splicing factor, and *R05D11.4* encodes an RNA helicase required for translation (Table 2). Thus, perturbing components of the gene expression machinery can enhance proteostasis, likely by altering the expression load of unstable proteins and activating stress responses to restore proteostasis. In particular, knockdown of *Y110A7A.8* activates the osmotic stress response [69] and causes premature onset of polyQ aggregation on 3 day old animals [35], consistent with an increase in misfolding (Table S3). However, on 6 day old animals we show that the number of aggregates is suppressed, which suggests a time-dependent response by the PN to enhance the folding machinery and restore folding. *gei-11* encodes a Myb-family transcription factor proposed to regulate cholinergic receptor function at the BWM cells [72], which affects muscle function and homeostasis [43], [73]; and *let-607* encodes a CREBH ortholog transcription factor predicted to be a component of the *C. elegans* ER stress response. The role of *let-607* is particularly intriguing as it reveals a genetic crosstalk between the cytoplasmic and ER lumen stress pathways. Knockdown of *let-607* induces chaperone expression that is not dependent upon HSF-1 (Figure 7B, 7C), suggesting that other stress responses such as the ER unfolded protein response may be involved in the suppression of cytosolic protein aggregation. Taken together, these results emphasize that diverse genetic and cellular mechanisms can restore cellular proteostasis beyond the traditional heat shock response. These nine gene modifiers of BWM protein homeostasis represent core components of the PN that evoke a robust and effective improvement of disease-related and endogenous metastable protein folding. Identification of these processes is a fundamental step towards identifying new components that constitute the network, and the cellular and organismal mechanisms by which they contribute to protein homeostasis and protect against chronic expression of misfolded toxic proteins.

## Materials and Methods

### *C. elegans* Strains and Maintenance

Animals were maintained according to standard methods, at 20°C on nematode growth media (NGM) with OP50 *E. coli* [74]. The strains utilized in this work, and previously described, are the following: wild-type (wt) Bristol strain N2; polyQ strains Q0 AM134 (*rmIs126[P_unc-54_::yfp]*), Q24 AM138 (*rmIs130[P_unc-54_::q24::yfp]II*), Q35 AM140 (*rmIs132[P_unc-54_::q35::yfp]I*), Q37 AM470 (*rmIs225[P_unc-54_::q37::yfp]II*) (Text S1) [26], [35]; human SOD1 strains SOD1^G93A^ AM265 (*rmIs177[P_unc-54_::sod1^G93A^::yfp]*) and SOD1^WT^ AM263 (*rmIs175[P_unc-54_::sod1^wt^::yfp]*) [12]; temperature sensitive (TS) mutant strains CB1402 [*unc-15(e1402)*], CB1157 [*unc-54(e1157)*], HE250 [*unc-52(e669su250)*] and CB286 [*unc-45(e286)*] [10]. The transgenic polyQ and SOD1 strains had been integrated by gamma-irradiation, 5 times backcrossed, and were previously described [12], [26]. The strain Q37;*hsf-1(sy441)* was generated by genetic cross of the original strains AM470 and PS3551[*hsf-1(sy441)*]I.

### RNA Interference Screen

The genome-wide RNAi screen for suppression of aggregation in *C. elegans* muscle cells was performed using the commercial RNAi library, with bacteria expressing dsRNA for 87% of the predicted *C. elegans* genes (GeneService, USA) [35], [40]. A semi-automated high throughput setup system was used, consisting of a robotic device (Biomek FX Liquid Handler, Beckman Coulter, USA) programmed to add bacteria and age-synchronized animals in liquid culture to 96-well plates. RNAi bacterial cultures were grown for approximately 8 h in LB-ampicillin 50 µg/ml (65 µl), at 37°C with continuous shaking at 315 rpm (Orbital shaker, GeneMachines HiGro, Genomic Solutions, USA), and induced with 0.5 mM isopropyl β-D-thiogalatoside (IPTG, Sigma) for 3 h at 37°C. To obtain an age synchronized population of L1 larvae (first larval state post egg hatching), Q35 gravid adults were bleached with a NaOCl solution [250 mM NaOH and 1∶4 (v/v) dilution of commercial bleach] and the eggs hatched in M9 buffer overnight at 20°C. Day 1 is defined as 18 h following NaOCl age-synchronization and animals are said to be 1 day old (L1 stage). 10 to 15 animals were added to each well in the 96-well plate in a volume of 50 µl of M9 plus [M9, 1 µg/ml cholesterol, 50 µg/ml ampicillin, 10 µg/ml tetracycline, 0.1 µg/ml fungizone and 170 µg/ml IPTG] and incubated at 20°C with continuous shaking at 200 rpm (Innova 4430 Incubator Shaker, New Brunswick, USA). Animals were scored 5 days later (6 days old) for reduction in the number of fluorescent foci using the stereomicroscope Leica MZ16FA equipped for epifluorescence (Leica Microsystems, Switzerland). As a negative control, animals were fed bacteria carrying the L4440 empty vector (EV). Suppression of aggregation was scored positive when more than 50% of the animals had a 50% or higher reduction in foci number relative to the EV control, without loss of YFP fluorescence, changes in growth rate or development of the animals. The candidate positive hits were re-screened (n≥3), then tested in the Q24 soluble control strain, and counter screened in Q37 animals (5 days old) and SOD1^G93A^ animals (5 days old). In Q37 and SOD1^G93A^ animals, suppression of aggregation was scored positive when more than 50% of the animals showed a reduction in foci number (>25%). RNAi was always added on day 1. The identity of the RNAi-targeted genes was verified by sequencing of the dsRNA plasmids, followed by Blast analysis in the NCBI and Wormbase databases revealing high specificity of genomic sequence targeting. Gene-knockdown by the respective RNAi was also confirmed for a representative group of hits by rtPCR (data not shown). For RNAi assays on plates (for foci scoring, FRAP and motility analysis, to collect animals for western blot and real-time qPCR, and for TS assays), NGM media was supplemented with 100 µg/ml ampicillin, 1 mM IPTG and 12 µg/ml tetracycline (Sigma), and seeded with overnight (16 h) RNAi bacteria cultures, pre-induced with IPTG (1 mM, 3 h). One day old (L1) animals (15 to 20 animals) were transferred onto NGM-RNAi bacteria seeded plates and grown at 20°C, and at the time indicated aggregation was scored in at least 50 animals, for each condition (n = 3). Aggregates were defined as discrete, bright foci that can be distinguished from their surrounding fluorescence by increased brightness intensity. The detection limit for these foci, measured with the higher resolution Zeiss Axiovert 200 microscope, is in the order of 3 µm in length (for elongated foci in Q35) and ∼7 µm^2^ in area (for round foci), with the microscopy tools and fluorescence exposure utilized in the genetic screen (Leica MZ16FA). Data collected from different experiments was compiled to calculate aggregate number averages relative to the control in EV RNAi. Fluorescent microscopy images were taken using an Axiovert 200 microscope with a Hamamatsu digital camera C4742-98 (Carl Zeiss, Germany). All assays were performed blind as to the identity of the RNAi by attributing to each modifier a number corresponding to a well with the dsRNA bacterial stock, in a 96-well plate.

### Fluorescence Recovery after Photobleaching Analysis

To examine the biophysical properties of polyQ protein, animals were subjected to FRAP analysis. Animals were mounted on a 3% (w/v) agar pad on a glass slide and immobilized in 2 mM levamisole. FRAP was measured using the Zeiss LSM510 confocal microscope (Carl Zeiss, Germany), and the 63× objective lens at 5× zoom power, with the 514 nm line for excitation. An area of 0.623 µm^2^ was bleached for 35 iterations at 100% transmission, after which time an image was collected every 123.35 ms. Relative fluorescence intensity (RFI) was determined as previously described [43], [75].

### SDS-PAGE, Native-PAGE, and Western Blotting Analysis

For SDS-PAGE analysis, 6 day old animals grown on RNAi-seeded NGM plates were collected and resuspended in PELE buffer [20 mM Tris pH7.4, 10% glycerol, 2% Triton X-100, 0.5 mM PMSF, 1 µg/ml leupeptin, 1 µg/ml pepstatin, 1 mM EDTA, 1 mM DTT, protease inhibitor cocktail tablet (Roche Diagnostics #11836170001)]. Lysis of ∼100 animals was accomplished by a combination of 4 cycles of freeze-thaw, grinding with a motorized pestle (Kontes #749541-000 and #749520-0000), followed by 8 min sonication (Sonicator Bath Branson 1510, Branson). To dissolve the polyQ aggregates, SDS was added to a final concentration of 5.5% (v/v) and samples were boiled for a total of 10 min. Total protein concentration was determined using the Bradford assay (Bio-Rad #500-0006). 15 µg (for Q35) or 20 µg (for SOD1) of total protein, in the linear range for YFP detection [43], were analyzed on a 10% SDS-PAGE followed by Western blotting. For YFP (polyQ and SOD1) detection, blots were probed with the anti-GFP IR800 conjugated antibody (1∶5,000 dilution; Rockland Immunochemicals #600-132-215). For α-tubulin detection, blots were probed with the anti-α-tubulin primary antibody (1;4,000 dilution; Sigma #T-5168) followed by the secondary antibody Alexa Fluor 680 goat anti-mouse IgG (1∶10,000 dilution; Molecular probes #A-21057). Antibody binding was detected with the Odyssey Infrared Imaging System (LI-COR Biosciences, USA). The ratio between band intensities YFP/α-tubulin was calculated for each sample (Adobe Photoshop 7.0, arbitrary units) and compared to the EV control (relative %). A representative group of modifiers was tested (3 biological replicates). Statistically significant changes in protein amounts were considered if *p<0.05* (Student's T-test).

For native PAGE analysis, animals (∼100) were collected with M9 buffer and resuspended in native-lysis buffer [50 mM Tris pH7.4, 5 mM MgCl_2_, 0.5% Triton X-100, 0.2 mM PMSF, 1 µg/ml leupeptin, protease inhibitor cocktail tablet (Roche Diagnostics #11836170001)]. Lysis was achieved with 4 cycles of freeze-thaw, and homogenization by grinding with the motorized pestle, always maintaining the tubes on ice. Total protein concentration was determined as before and 40 µg were analyzed on a 5% native PAGE (at 4°C), followed by gel scan (STORM 860, #91393, GE Healthcare, UK). This experiment was done in triplicate.

### Motility Assays

Animals (6 days old) grown on RNAi NGM plates at 20°C were picked (20–25 animals) onto the center of a NGM OP50-seeded plate (full surface area covered with OP50), equilibrated at 20°C. Animals' movements were digitally recorded using a Leica M205 FA microscope with a Hamamatsu digital camera C10600-10B (Orca-R2, Leica Microsystems, Switzerland), and the Hamamatsu Simple PCI Imaging software. Videos of 45 s were recorded at 2×2 binning and 5 frames per second, and captured frames were merged into *.avi format and imported directly into ImageJ. Using the LOCI bio-formats plugin and a custom stack de-flicker plugin (http://www.loci.wisc.edu/bio-formats/imagej), light average intensity was normalized for each frame. To enhance the definition of the animals in the movies, the difference between each frame and the constant background was calculated, using the ‘Maximum Z-stack’ projection. The resulting movie was converted to binary format using Otsu Thresholding 2. Binary objects representing the animals were tracked using custom ImageJ plugin, wrMTrck (based on “MTrack2” by Nico Stuurman [76]). The average speed of each animal was calculated by dividing the length of each track (corrected for animal body length) by the duration of the track (body length per second, or BLPS). The wrMTrck plugin and scripts for automated analysis are open-source and publicly available at http://www.phage.dk/plugins. Videos were recorded for a minimum of 75 animals per experiment (*n*≥3) and motility measurements are given as a percentage of wt motility (% wt in EV RNAi). RNAi modifiers that affected the motility of wt animals were removed from further analysis. All motility assays were also performed blind as to the identity of the RNAi gene-target. Measurements of motility were validated by other read-outs that included manual-based motility assays [10], [77]. The first manual assay measured how fast it took animals placed in the center of a ring (circumference only) of OP50 bacteria to reach the food, and the second manual assay monitored the number of worms that traveled 1 cm in 1 minute on OP50 bacteria-seeded NGM plates. All results shown were obtained with the automated worm tracker, which provides reproducible and unbiased results.

### Assay for TS Phenotypes

Temperature sensitive (TS) mutant animals were age-synchronized to L1 stage by NaOCl bleaching, grown on RNAi-seeded NGM plates (15–20 animals per plate) from day 1 at a sensitized temperature of 23°C (to maintain the RNAi suppressor effect on aggregation, which was used as a control) and scored for phenotypes on day 5. For the 25°C restrictive temperature control experiment, L1 nematodes were grown on EV RNAi at 15°C until L4 stage to avoid embryonic and developmental phenotypes, then transferred to 25°C and scored 2 days later for the same phenotypes. For the 15°C permissive temperature control experiment, animals were synchronized to L1, added to EV RNAi plates, grown at 15°C and scored for phenotypes on day 6 (to account for slower but normal development at this temperature). At least 50 animals were scored for each specific phenotype, per experiment (*n = 3*), as described previously [10], [42], [43], and all assays were performed blind. For the slow movement/paralysis assay [*unc-15(e1402)* and *unc-54(e1157)*], 15–20 animals were placed on a OP50-NGM plate at room temperature in the center of a 1 cm circle (drawn on the bottom of the plate). Animals remaining in the 1 cm circle after 5 min were considered to possess a slow movement or paralyzed phenotype. To score for stiff paralysis [*unc-52(e669su250)*], partially paralyzed animals with moving heads and stick-like bodies were scored. For the egg-laying phenotype [*unc-45(e286)*] partially paralyzed animals with a large belly of accumulated eggs were scored.

### Real-Time qPCR

Wt animals (5 days old and ∼50) were collected from RNAi-NGM plates and RNA was extracted with the Trizol reagent (Invitrogen), followed by DNase treatment (Applied Biosystems #AM1906). mRNA was then reverse transcribed using the iScript cDNA Synthesis Kit (Bio-Rad #170-8891). 10 ng of cDNA were used for real-time PCR amplification using the iQ SYBR Green Supermix (Bio-Rad #170-8880) and the iCycler system (Bio-Rad) (see Text S1). The relative expression levels of each gene were determined using the Comparative C_T_ Method (Real-Time PCR Applications Guide, Bio-Rad). Gene expression levels were normalized relative to actin (*act-1*) in the same sample (internal control), and then relative to the levels of the same gene in EV control sample. Measurements were performed for ≥3 biological samples for each condition.

## Supporting Information

Figure S1 *C. elegans* models of polyQ and human SOD1 aggregation. (A) Q35 and Q37 body sections show distinct morphology of aggregates (white arrows) in 6 and 5 day old animals, respectively. Scale bar is 0.1 mm. (B) Time dependent aggregate count for Q35 and Q37 animals (±SD, n>3). (C) SDS-PAGE and western blot analysis of protein samples from animals (6 days old) expressing polyQ-YFP protein, immunoblotted with anti-YFP (top) and anti-α-tubulin (bottom) antibodies. By standard protein extraction, Q35 and Q37 aggregates are SDS-insoluble and are trapped in the loading well. YFP/tubulin ratios were calculated from protein band intensities (total YFP) and are shown relative to Q0 (±SD). (D) Motility measurements (in body length per second/BLPS) of 6 day old wt and polyQ animals show that Q35 and Q37 aggregation in BWM cells causes a motility defect (±SEM, n = 3, Student t-test ***p<0.001). (E,F) Expression of human SOD1-YFP in muscle cells: while SOD1^wt^ adopts a diffuse soluble fluorescent pattern (E: II and III are zoom in of the boxed areas on I), mutant SOD1^G93A^ displays a pattern of small foci (F: II and III are zoom in of the boxed areas on I). Scale bar is 0.1 mm.
(TIF)

Figure S2 PolyQ mRNA levels in RNAi treated animals. *q35-yfp* mRNA levels from RNAi-treated animals (6 days old) analyzed by reverse transcriptase PCR amplification (top). Control corresponds to EV and *yfp*-RNAi is the positive control for reduced *q35-yfp* mRNA levels. Actin mRNA (bottom) is the control for total mRNA levels. Ratio *q35-yfp/actin* are calculated from band intensities, and averaged from 3 biological replicates. Student t-test *p*>0.05 for all but *yfp* (***p<0.001).
(TIF)

Figure S3 Gene modifiers of aggregation distribution into functional classes. (A) *C. elegans* genes represented in the RNAi library (16,757). (B) RNAi suppressors of Q35 aggregation (151). (C) Common aggregation suppressors for polyQ and SOD1^G93A^ (63). Statistical significance calculated by the Chi-square test ***p<0.001.
(TIF)

Table S1 Exhaustive list and description of all RNAi targeted genes that suppressed Q35 aggregation.
(XLS)

Table S2 Motility assays results for each modifier tested, per assay executed, for wt and Q35 animals.
(XLS)

Table S3 Comparative analysis between genetic screens, and overlapping gene modifiers.
(DOC)

Text S1 Supporting Information: Materials and Methods.
(DOC)

## References

[1] Taylor JP, Hardy J, Fischbeck KH (2002). Toxic proteins in neurodegenerative disease.. Science.

[2] Ruegg MA, Glass DJ (2011). Molecular mechanisms and treatment options for muscle wasting diseases.. Annual review of pharmacology and toxicology.

[3] Soto C (2003). Unfolding the role of protein misfolding in neurodegenerative diseases.. Nat Rev Neurosci.

[4] Soto C, Estrada LD (2008). Protein misfolding and neurodegeneration.. Arch Neurol.

[5] Stefani M (2004). Protein misfolding and aggregation: new examples in medicine and biology of the dark side of the protein world.. Biochim Biophys Acta.

[6] Balch WE, Morimoto RI, Dillin A, Kelly JW (2008). Adapting proteostasis for disease intervention.. Science.

[7] Morimoto RI (2008). Proteotoxic stress and inducible chaperone networks in neurodegenerative disease and aging.. Genes Dev.

[8] Morimoto RI, Cuervo AM (2009). Protein homeostasis and aging: taking care of proteins from the cradle to the grave.. The journals of gerontology Series A, Biological sciences and medical sciences.

[9] Olzscha H, Schermann SM, Woerner AC, Pinkert S, Hecht MH (2011). Amyloid-like aggregates sequester numerous metastable proteins with essential cellular functions.. Cell.

[10] Gidalevitz T, Ben-Zvi A, Ho KH, Brignull HR, Morimoto RI (2006). Progressive disruption of cellular protein folding in models of polyglutamine diseases.. Science.

[11] Gidalevitz T, Kikis EA, Morimoto RI (2010). A cellular perspective on conformational disease: the role of genetic background and proteostasis networks.. Curr Opin Struct Biol.

[12] Gidalevitz T, Krupinski T, Garcia S, Morimoto RI (2009). Destabilizing protein polymorphisms in the genetic background direct phenotypic expression of mutant SOD1 toxicity.. PLoS Genet.

[13] Ross CA, Poirier MA (2005). Opinion: What is the role of protein aggregation in neurodegeneration?. Nat Rev Mol Cell Biol.

[14] Treusch S, Cyr DM, Lindquist S (2009). Amyloid deposits: protection against toxic protein species?. Cell cycle.

[15] Arrasate M, Mitra S, Schweitzer ES, Segal MR, Finkbeiner S (2004). Inclusion body formation reduces levels of mutant huntingtin and the risk of neuronal death.. Nature.

[16] Gutekunst CA, Li SH, Yi H, Mulroy JS, Kuemmerle S (1999). Nuclear and neuropil aggregates in Huntington's disease: relationship to neuropathology.. J Neurosci.

[17] Kirkitadze MD, Bitan G, Teplow DB (2002). Paradigm shifts in Alzheimer's disease and other neurodegenerative disorders: the emerging role of oligomeric assemblies.. J Neurosci Res.

[18] Van Raamsdonk JM, Murphy Z, Slow EJ, Leavitt BR, Hayden MR (2005). Selective degeneration and nuclear localization of mutant huntingtin in the YAC128 mouse model of Huntington disease.. Hum Mol Genet.

[19] Zoghbi HY, Orr HT (1999). Polyglutamine diseases: protein cleavage and aggregation.. Current opinion in neurobiology.

[20] Glabe CG, Kayed R (2006). Common structure and toxic function of amyloid oligomers implies a common mechanism of pathogenesis.. Neurology.

[21] van Ham TJ, Breitling R, Swertz MA, Nollen EA (2009). Neurodegenerative diseases: Lessons from genome-wide screens in small model organisms.. EMBO molecular medicine.

[22] Powers ET, Morimoto RI, Dillin A, Kelly JW, Balch WE (2009). Biological and chemical approaches to diseases of proteostasis deficiency.. Annu Rev Biochem.

[23] Brignull HR, Moore FE, Tang SJ, Morimoto RI (2006). Polyglutamine proteins at the pathogenic threshold display neuron-specific aggregation in a pan-neuronal Caenorhabditis elegans model.. J Neurosci.

[24] Faber PW, Alter JR, MacDonald ME, Hart AC (1999). Polyglutamine-mediated dysfunction and apoptotic death of a Caenorhabditis elegans sensory neuron.. Proc Natl Acad Sci U S A.

[25] Feany MB, Bender WW (2000). A Drosophila model of Parkinson's disease.. Nature.

[26] Morley JF, Brignull HR, Weyers JJ, Morimoto RI (2002). The threshold for polyglutamine-expansion protein aggregation and cellular toxicity is dynamic and influenced by aging in Caenorhabditis elegans.. Proc Natl Acad Sci U S A.

[27] Outeiro TF, Muchowski PJ (2004). Molecular genetics approaches in yeast to study amyloid diseases.. J Mol Neurosci.

[28] Van Raamsdonk JM, Warby SC, Hayden MR (2007). Selective degeneration in YAC mouse models of Huntington disease.. Brain Res Bull.

[29] Warrick JM, Paulson HL, Gray-Board GL, Bui QT, Fischbeck KH (1998). Expanded polyglutamine protein forms nuclear inclusions and causes neural degeneration in Drosophila.. Cell.

[30] Watson MR, Lagow RD, Xu K, Zhang B, Bonini NM (2008). A Drosophila model for amyotrophic lateral sclerosis reveals motor neuron damage by human SOD1.. J Biol Chem.

[31] Kraemer BC, Zhang B, Leverenz JB, Thomas JH, Trojanowski JQ (2003). Neurodegeneration and defective neurotransmission in a Caenorhabditis elegans model of tauopathy.. Proceedings of the National Academy of Sciences of the United States of America.

[32] Bilen J, Bonini NM (2007). Genome-wide screen for modifiers of ataxin-3 neurodegeneration in Drosophila.. PLoS Genet.

[33] Kazemi-Esfarjani P, Benzer S (2000). Genetic suppression of polyglutamine toxicity in Drosophila.. Science.

[34] Kraemer BC, Burgess JK, Chen JH, Thomas JH, Schellenberg GD (2006). Molecular pathways that influence human tau-induced pathology in Caenorhabditis elegans.. Hum Mol Genet.

[35] Nollen EA, Garcia SM, van Haaften G, Kim S, Chavez A (2004). Genome-wide RNA interference screen identifies previously undescribed regulators of polyglutamine aggregation.. Proc Natl Acad Sci U S A.

[36] van Ham TJ, Thijssen KL, Breitling R, Hofstra RM, Plasterk RH (2008). C. elegans model identifies genetic modifiers of alpha-synuclein inclusion formation during aging.. PLoS Genet.

[37] Wang J, Farr GW, Hall DH, Li F, Furtak K (2009). An ALS-linked mutant SOD1 produces a locomotor defect associated with aggregation and synaptic dysfunction when expressed in neurons of Caenorhabditis elegans.. PLoS Genet.

[38] Willingham S, Outeiro TF, DeVit MJ, Lindquist SL, Muchowski PJ (2003). Yeast genes that enhance the toxicity of a mutant huntingtin fragment or alpha-synuclein.. Science.

[39] Zhang S, Binari R, Zhou R, Perrimon N (2010). A genomewide RNA interference screen for modifiers of aggregates formation by mutant Huntingtin in Drosophila.. Genetics.

[40] Kamath RS, Ahringer J (2003). Genome-wide RNAi screening in Caenorhabditis elegans.. Methods.

[41] Kamath RS, Fraser AG, Dong Y, Poulin G, Durbin R (2003). Systematic functional analysis of the Caenorhabditis elegans genome using RNAi.. Nature.

[42] Ben-Zvi A, Miller EA, Morimoto RI (2009). Collapse of proteostasis represents an early molecular event in Caenorhabditis elegans aging.. Proc Natl Acad Sci U S A.

[43] Garcia SM, Casanueva MO, Silva MC, Amaral MD, Morimoto RI (2007). Neuronal signaling modulates protein homeostasis in Caenorhabditis elegans post-synaptic muscle cells.. Genes Dev.

[44] Gengyo-Ando K, Kagawa H (1991). Single charge change on the helical surface of the paramyosin rod dramatically disrupts thick filament assembly in Caenorhabditis elegans.. Journal of molecular biology.

[45] Dillin A, Hsu AL, Arantes-Oliveira N, Lehrer-Graiwer J, Hsin H (2002). Rates of behavior and aging specified by mitochondrial function during development.. Science.

[46] Lee SS, Lee RY, Fraser AG, Kamath RS, Ahringer J (2003). A systematic RNAi screen identifies a critical role for mitochondria in C. elegans longevity.. Nat Genet.

[47] Rea SL, Ventura N, Johnson TE (2007). Relationship between mitochondrial electron transport chain dysfunction, development, and life extension in Caenorhabditis elegans.. PLoS Biol.

[48] Durieux J, Wolff S, Dillin A (2011). The cell-non-autonomous nature of electron transport chain-mediated longevity.. Cell.

[49] Hansen M, Taubert S, Crawford D, Libina N, Lee SJ (2007). Lifespan extension by conditions that inhibit translation in Caenorhabditis elegans.. Aging Cell.

[50] Tavernarakis N (2008). Ageing and the regulation of protein synthesis: a balancing act?. Trends Cell Biol.

[51] Mattson MP (2008). Dietary factors, hormesis and health.. Ageing Res Rev.

[52] Muchowski PJ, Schaffar G, Sittler A, Wanker EE, Hayer-Hartl MK (2000). Hsp70 and hsp40 chaperones can inhibit self-assembly of polyglutamine proteins into amyloid-like fibrils.. Proc Natl Acad Sci U S A.

[53] Sakahira H, Breuer P, Hayer-Hartl MK, Hartl FU (2002). Molecular chaperones as modulators of polyglutamine protein aggregation and toxicity.. Proc Natl Acad Sci U S A.

[54] Wang X, Venable J, LaPointe P, Hutt DM, Koulov AV (2006). Hsp90 cochaperone Aha1 downregulation rescues misfolding of CFTR in cystic fibrosis.. Cell.

[55] Cummings CJ, Sun Y, Opal P, Antalffy B, Mestril R (2001). Over-expression of inducible HSP70 chaperone suppresses neuropathology and improves motor function in SCA1 mice.. Hum Mol Genet.

[56] Freeman BC, Toft DO, Morimoto RI (1996). Molecular chaperone machines: chaperone activities of the cyclophilin Cyp-40 and the steroid aporeceptor-associated protein p23.. Science.

[57] Evans CG, Wisen S, Gestwicki JE (2006). Heat shock proteins 70 and 90 inhibit early stages of amyloid beta-(1–42) aggregation in vitro.. J Biol Chem.

[58] Wacker JL, Zareie MH, Fong H, Sarikaya M, Muchowski PJ (2004). Hsp70 and Hsp40 attenuate formation of spherical and annular polyglutamine oligomers by partitioning monomer.. Nat Struct Mol Biol.

[59] Ballinger CA, Connell P, Wu Y, Hu Z, Thompson LJ (1999). Identification of CHIP, a novel tetratricopeptide repeat-containing protein that interacts with heat shock proteins and negatively regulates chaperone functions.. Mol Cell Biol.

[60] Nollen EA, Brunsting JF, Song J, Kampinga HH, Morimoto RI (2000). Bag1 functions in vivo as a negative regulator of Hsp70 chaperone activity.. Mol Cell Biol.

[61] Muchowski PJ, Ning K, D'Souza-Schorey C, Fields S (2002). Requirement of an intact microtubule cytoskeleton for aggregation and inclusion body formation by a mutant huntingtin fragment.. Proc Natl Acad Sci U S A.

[62] Legleiter J, Mitchell E, Lotz GP, Sapp E, Ng C (2010). Mutant huntingtin fragments form oligomers in a polyglutamine length-dependent manner in vitro and in vivo.. The Journal of biological chemistry.

[63] Takahashi Y, Okamoto Y, Popiel HA, Fujikake N, Toda T (2007). Detection of polyglutamine protein oligomers in cells by fluorescence correlation spectroscopy.. J Biol Chem.

[64] Thakur AK, Jayaraman M, Mishra R, Thakur M, Chellgren VM (2009). Polyglutamine disruption of the huntingtin exon 1 N terminus triggers a complex aggregation mechanism.. Nat Struct Mol Biol.

[65] Miller J, Arrasate M, Shaby BA, Mitra S, Masliah E (2010). Quantitative relationships between huntingtin levels, polyglutamine length, inclusion body formation, and neuronal death provide novel insight into huntington's disease molecular pathogenesis.. The Journal of neuroscience: the official journal of the Society for Neuroscience.

[66] Cohen E, Bieschke J, Perciavalle RM, Kelly JW, Dillin A (2006). Opposing activities protect against age-onset proteotoxicity.. Science.

[67] Karpinar DP, Balija MB, Kugler S, Opazo F, Rezaei-Ghaleh N (2009). Pre-fibrillar alpha-synuclein variants with impaired beta-structure increase neurotoxicity in Parkinson's disease models.. The EMBO journal.

[68] Saudou F, Finkbeiner S, Devys D, Greenberg ME (1998). Huntingtin acts in the nucleus to induce apoptosis but death does not correlate with the formation of intranuclear inclusions.. Cell.

[69] Lamitina T, Huang CG, Strange K (2006). Genome-wide RNAi screening identifies protein damage as a regulator of osmoprotective gene expression.. Proc Natl Acad Sci U S A.

[70] Hamilton B, Dong Y, Shindo M, Liu W, Odell I (2005). A systematic RNAi screen for longevity genes in C. elegans.. Genes Dev.

[71] Giorgini F, Guidetti P, Nguyen Q, Bennett SC, Muchowski PJ (2005). A genomic screen in yeast implicates kynurenine 3-monooxygenase as a therapeutic target for Huntington disease.. Nat Genet.

[72] Gottschalk A, Almedom RB, Schedletzky T, Anderson SD, Yates JR (2005). Identification and characterization of novel nicotinic receptor-associated proteins in Caenorhabditis elegans.. EMBO J.

[73] Szewczyk NJ, Hartman JJ, Barmada SJ, Jacobson LA (2000). Genetic defects in acetylcholine signalling promote protein degradation in muscle cells of Caenorhabditis elegans.. J Cell Sci.

[74] Brenner S (1974). The genetics of Caenorhabditis elegans.. Genetics.

[75] Phair RD, Misteli T (2000). High mobility of proteins in the mammalian cell nucleus.. Nature.

[76] Klopfenstein DR, Vale RD (2004). The lipid binding pleckstrin homology domain in UNC-104 kinesin is necessary for synaptic vesicle transport in Caenorhabditis elegans.. Mol Biol Cell.

[77] Teixeira-Castro A, Ailion M, Jalles A, Brignull HR, Vilaca JL (2011). Neuron-specific proteotoxicity of mutant ataxin-3 in C. elegans: rescue by the DAF-16 and HSF-1 pathways.. Human molecular genetics.

