# Supplementary figures and images for: A Genetic Screening Strategy Identifies Novel Regulators of the Proteostasis Network
Source: PLoS Genet. 2011 Dec 29;7(12):e1002438. doi: 10.1371/journal.pgen.1002438 (PMC3248563; doi:10.1371/journal.pgen.1002438)

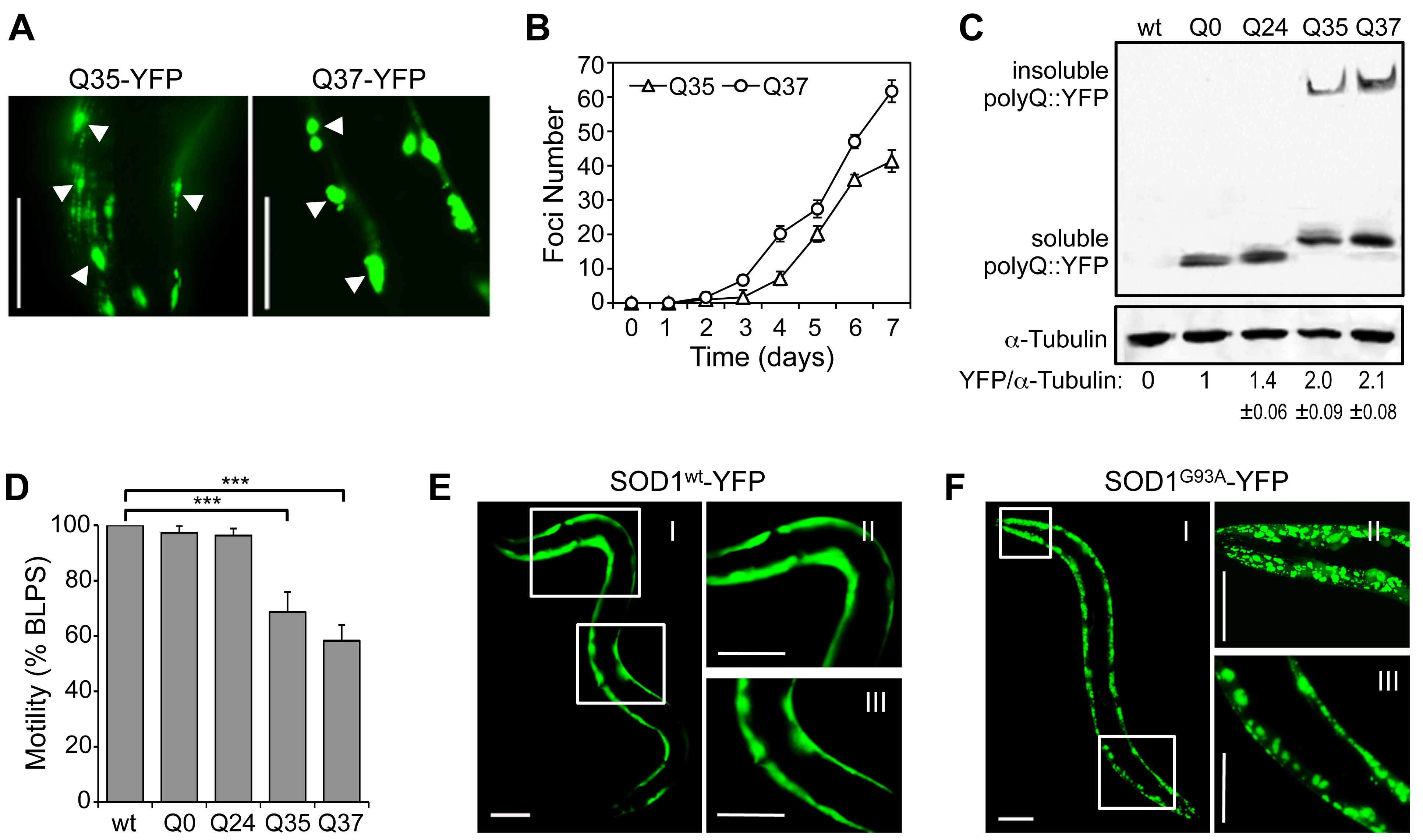

Supplement: Figure S1 — C. elegans models of polyQ and human SOD1 aggregation. (A) Q35 and Q37 body sections show distinct morphology of aggregates (white arrows) in 6 and 5 day old animals, respectively. Scale bar is 0.1 mm. (B) Time dependent aggregate count for Q35 and Q37 animals (±SD, n>3). (C) SDS-PAGE and western blot analysis of protein samples from animals (6 days old) expressing polyQ-YFP protein, immunoblotted with anti-YFP (top) and anti-α-tubulin (bottom) antibodies. By standard protein extraction, Q35 and Q37 aggregates are SDS-insoluble and are trapped in the loading well. YFP/tubulin ratios were calculated from protein band intensities (total YFP) and are shown relative to Q0 (±SD). (D) Motility measurements (in body length per second/BLPS) of 6 day old wt and polyQ animals show that Q35 and Q37 aggregation in BWM cells causes a motility defect (±SEM, n = 3, Student t-test ***p<0.001). (E,F) Expression of human SOD1-YFP in muscle cells: while SOD1wt adopts a diffuse soluble fluorescent pattern (E: II and III are zoom in of the boxed areas on I), mutant SOD1G93A displays a pattern of small foci (F: II and III are zoom in of the boxed areas on I). Scale bar is 0.1 mm. (TIF) [file pgen.1002438.s001.tif]

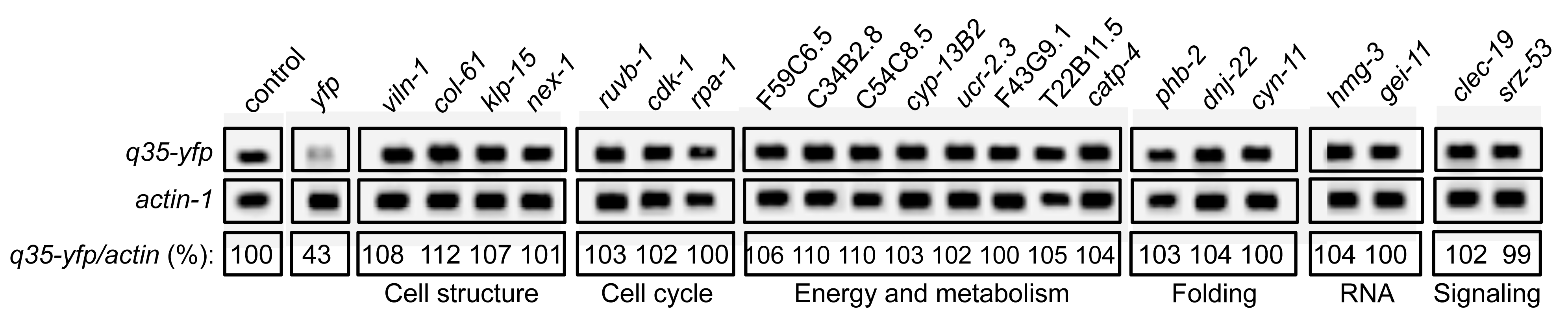

Supplement: Figure S2 — PolyQ mRNA levels in RNAi treated animals. q35-yfp mRNA levels from RNAi-treated animals (6 days old) analyzed by reverse transcriptase PCR amplification (top). Control corresponds to EV and yfp-RNAi is the positive control for reduced q35-yfp mRNA levels. Actin mRNA (bottom) is the control for total mRNA levels. Ratio q35-yfp/actin are calculated from band intensities, and averaged from 3 biological replicates. Student t-test p>0.05 for all but yfp (***p<0.001). (TIF) [file pgen.1002438.s002.tif]

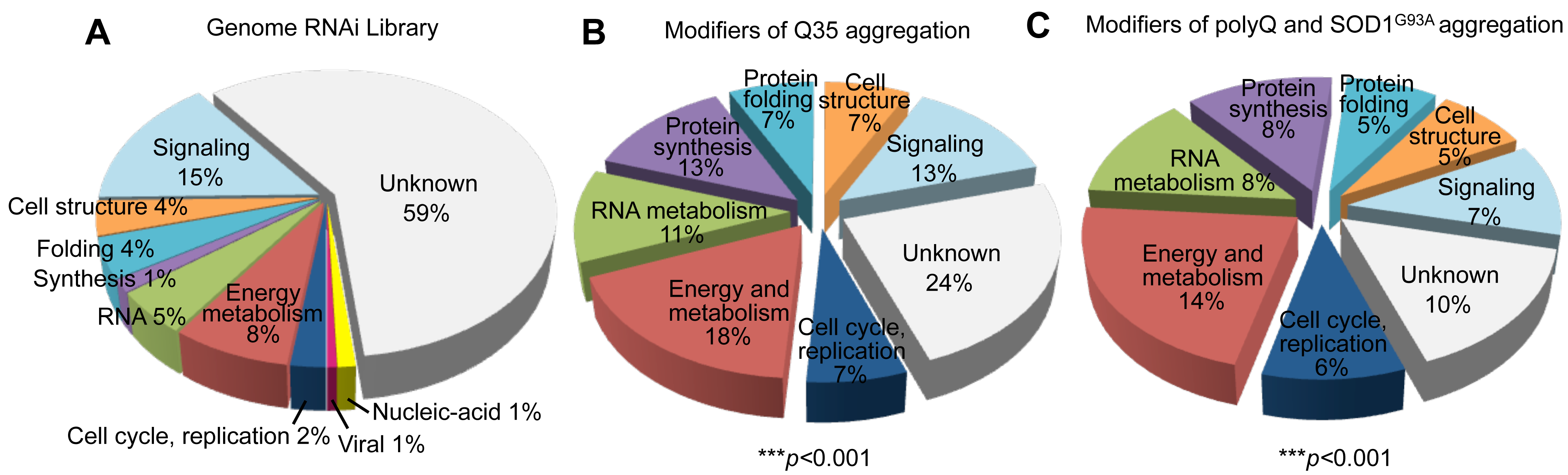

Supplement: Figure S3 — Gene modifiers of aggregation distribution into functional classes. (A) C. elegans genes represented in the RNAi library (16,757). (B) RNAi suppressors of Q35 aggregation (151). (C) Common aggregation suppressors for polyQ and SOD1G93A (63). Statistical significance calculated by the Chi-square test ***p<0.001. (TIF) [file pgen.1002438.s003.tif]
