# Supplementary material for: A Genetic Screening Strategy Identifies Novel Regulators of the Proteostasis Network
Source: PLoS Genet. 2011 Dec 29;7(12):e1002438. doi: 10.1371/journal.pgen.1002438 (PMC3248563; doi:10.1371/journal.pgen.1002438)
Supplement: Table S3 — Comparative analysis between genetic screens, and overlapping gene modifiers. (DOC) [file pgen.1002438.s006.doc]

**Table S3:** Comparative analysis between genetic screens and overlapping gene modifiers.

| **Genetic Screen** | **Overlap with** | **Other genes in common with** |
| --- | --- | --- |
| **(Author, year, screen, organism)** | **the final group** | **Q35 aggregation suppressor** |
|  | **of nine modifiers** | **modifiers** |
| **Hamilton B *et al.* 2005** | *F43G9.1* | - |
| (longevity; *C. elegans)* |  |  |
| **Hansen M *et al*. 2005** | - | *ril-1; C53A5.1* |
| (longevity; *C. elegans*) |  |  |
| **Murphy C *et al*. 2003** | - | *tps-2; mua-6; Y43C5A.3* |
| (DAF-16 downstream targets; *C. elegans*) |  |  |
| **Wang J *et al*. 2009 (**neuronal SOD1^G85R^ | - | *catp-4; C30C11.4* |
| aggregation; *C. elegans)* |  |  |
| **van Ham T *et al.* 2008** | - | - |
| (α-synuclein aggregation; *C. elegans*) |  |  |
| **Kraemer B *et al.* 2006** | *ucr-2.3* | - |
| (tau toxicity; *C. elegans*) |  |  |
| **Lamitina T *et al.* 2006** | *Y110A7A.8* | *ruvb-1; krs-1; eif-3.B; C55A6.9* |
| (osmotic stress; *C. elegans*) |  |  |
| **Nollen E *et al.* 2004** | *Y110A7A.8* | *H19N07.1; rps-21; ril-1; rpl-2; rpl-23;* |
| (polyQ aggregation; *C. elegans*) |  | *rpl-35; rpl-7A; imb-3; T07A9.9* |
| **Zhang S *et al.* 2010** | *-* | *rpl-2; rpl-23; rpl-35; rpl-7A; imb-3;* |
| (Htt46Q aggregation; *Drosophila*) |  | *T07A9.9; C30C11.4* |
| **Bilen J *et al*. 2007** | - | *ZK742.1* |
| (ataxin toxicity/aggregation; *Drosophila*) |  |  |
| **Kazemi-Esfarjani P *et al.* 2000** | - | - |
| (polyQ toxicity; *Drosophila*) |  |  |
| **Willingham S *et al.* 2006** | - | - |
| (Htt53Q toxicity; Yeast) |  |  |
| **Giorgini F *et al.* 2005** | - | - |
| (Htt103Q toxicity; Yeast) |  |  |
| **Hodges A *et al*. 2006** (gene expression | - | - |
| changes in human HD brain) |  |  |
