# Supplementary material for: A Genetic Screening Strategy Identifies Novel Regulators of the Proteostasis Network
Source: PLoS Genet. 2011 Dec 29;7(12):e1002438. doi: 10.1371/journal.pgen.1002438 (PMC3248563; doi:10.1371/journal.pgen.1002438)
Supplement: Text S1 — Supporting Information: Materials and Methods. (DOC) [file pgen.1002438.s007.doc]

**Supporting Information**

**Materials and Methods**

**Q37-YFP Expression in *C. elegans* Muscle Cells**

The *unc-54*p::*q37*::*yfp* *C. elegans* strain, was generated as described before . The 37 CAG-repeats fused to YFP show a fluorescent diffuse pattern from hatching until day 2^1^/2 of age, after which small round foci start to appear (onset of aggregation) (Figure S1A, B). The foci correspond to aggregated SDS-insoluble protein retained at the top of the gel as shown by SDS-PAGE western blot analysis, when protein is extracted in native-like conditions (Figure S1C). Expression of Q37 in muscle cells disrupts animal motility by more than 40% relative to the wt or soluble control Q0 and Q24, indicating cellular dysfunction and toxicity (Figure S1D).

**Semi-Quantitative RT-PCR**

Six day old animals were collected for RNA extraction with the Trizol reagent (Invitrogen #15596-026). Lysis was accomplished by vortexing. Chloroform was added to each sample followed by vortexing and centrifugation (4°C). 2-Propanol was added to each aqueous layer and total RNA was spun down, washed with 75% (v/v) ethanol, air-dried and resuspended in nuclease free water. RNA aliquots of 10 µg were used for DNase treatment (Applied Biosystems #AM1906), and 1 µg purified RNA was used for cDNA synthesis (Bio-Rad #170-8891). cDNA samples were diluted to a final volume of 500 µl in water and 1 µl was used for PCR amplification with specific primers for the *q35-yfp* sequence (forward primer: CCTGGAGCATTTCCCCAC; reverse primer: GAACTTCAGGGTCAGCTTGCC) and actin (*act-1* forward primer: ATCACCGCTCTTGCCCCATC; reverse primer: GGCCGGACTCGTCGTATTCTTG). A representative group of 22 modifiers were tested. Band intensity ratios *q35-yfp/actin* were calculated with Adobe Photoshop 7.0 (arbitrary units) and are shown as relative % to the EV control.

**References**

1. Morley JF, Brignull HR, Weyers JJ, Morimoto RI (2002) The threshold for polyglutamine-expansion protein aggregation and cellular toxicity is dynamic and influenced by aging in Caenorhabditis elegans. Proc Natl Acad Sci U S A 99: 10417-10422.
